# Recent Advances of Field-Effect Transistor Technology for Infectious Diseases

**DOI:** 10.3390/bios11040103

**Published:** 2021-04-02

**Authors:** Abbas Panahi, Deniz Sadighbayan, Saghi Forouhi, Ebrahim Ghafar-Zadeh

**Affiliations:** 1 Biologically Sensors and Actuators (BioSA) Laboratory, Lassonde School of Engineering, York University, Keel Street, Toronto, ON M3J 1P3, Canada; panahiyu@yorku.ca (A.P.); denizsdg@yorku.ca (D.S.); sforouhi@yorku.ca (S.F.); 2 Department of Electrical Engineering and Computer Science, Lassonde School of Engineering, York University, Keel Street, Toronto, ON M3J 1P3, Canada; 3 Department of Biology, Faculty of Science, York University, Keel Street, Toronto, ON M3J 1P3, Canada

**Keywords:** field effect transistor, biosensor, infectious disease, COVID-19, label-free detection, CMOS-based readout circuit

## Abstract

Field-effect transistor (FET) biosensors have been intensively researched toward label-free biomolecule sensing for different disease screening applications. High sensitivity, incredible miniaturization capability, promising extremely low minimum limit of detection (LoD) at the molecular level, integration with complementary metal oxide semiconductor (CMOS) technology and last but not least label-free operation were amongst the predominant motives for highlighting these sensors in the biosensor community. Although there are various diseases targeted by FET sensors for detection, infectious diseases are still the most demanding sector that needs higher precision in detection and integration for the realization of the diagnosis at the point of care (PoC). The COVID-19 pandemic, nevertheless, was an example of the escalated situation in terms of worldwide desperate need for fast, specific and reliable home test PoC devices for the timely screening of huge numbers of people to restrict the disease from further spread. This need spawned a wave of innovative approaches for early detection of COVID-19 antibodies in human swab or blood amongst which the FET biosensing gained much more attention due to their extraordinary LoD down to femtomolar (fM) with the comparatively faster response time. As the FET sensors are promising novel PoC devices with application in early diagnosis of various diseases and especially infectious diseases, in this research, we have reviewed the recent progress on developing FET sensors for infectious diseases diagnosis accompanied with a thorough discussion on the structure of Chem/BioFET sensors and the readout circuitry for output signal processing. This approach would help engineers and biologists to gain enough knowledge to initiate their design for accelerated innovations in response to the need for more efficient management of infectious diseases like COVID-19.

## 1. Introduction

The recent decade has witnessed significant advancements in the detection and treatment of different diseases that have improved the level of human health globally. However, the COVID-19 pandemic taught us that we are not very well prepared for early diagnostic and management of infectious diseases at the time of pandemics for controlling the further spread of disease [1]. There are many hurdles in front of fast and resourceful management of infectious disease in the time of emergency like what happened during COVID-19 pandemics. The most noteworthy problems associated with current medical procedures for controlling infectious diseases are drug resistance evolution of pathogens and also the possibility of the appearance of new genetically developed agents, which might spread much faster than the previous virus case, as we saw an example of that during COVID-19 pandemic during which a newly evolved version of the virus was spotted in England and Africa. The newly evolved virus was potent in regard to being spread significantly faster into the population than the previous COVID-19 version [2,3]. As we see, the fast and accurate diagnosis along with proper timely treatment could considerably curb the further spread of the virus. Currently, there are very well-established laboratory-based techniques that are being used for screening and testing infectious diseases. These devices and gold standards are microscopies, culturing techniques, immunoassays, and PCR (Polymerase Chain Reaction) methods [4,5]. Notwithstanding the fact that these methods have been frequently used in fighting against many infectious diseases like sepsis, tuberculosis, human immunodeficiency virus (HIV), hepatitis, malaria, and so on, in the time of high demand for fast and highly accurate screening of population in pandemics, they have revealed shortages to meet those expectancies. Microscopy does not address the required high accuracy; immunoassays (e.g., enzyme-linked immunosorbent assay (ELISA)) are labour-intensive and not very well designed for multiplexing purposes. Furthermore, the PCR methods, despite being accurate at the molecular level, need tedious processes of sample preparation and experiment setup afterward. 

Apart from the equipment challenges and current state of the art, the process of sampling and sending them to standard machines in special laboratories have been recognized as added barriers in controlling the spread of diseases as it has escalated human interactions in the time of effective social distancing measures. This inefficient process has complicated the timely screening of the population and, as a result, lower than needed tests per day, which ends up with a huge number of people not screened. Consequently, this situation will cause more infected cases and lead to the further spread of the disease. This problem will be deteriorated in the countries with limited access to the standard equipment and advanced laboratories that cause the international spread of disease and will hamper the regular international connections.

Nevertheless, the recent decade was the booming time for the emergence of a wave of biosensing innovations toward the realization of early and timely detection of diseases through miniaturized devices with the anticipation of affordable-to-all handheld point-of-care (PoC) devices [5,6,7]. To better envisage the steps taken during this era of personalized medicine research and development, the glucose test is a good example to grasp fruitful endeavours. Glucose at-home tests are based on a potentiometric sensor and have been reported in different configurations [8]. These sensors have enabled the successful controlling and monitoring of diabetes disease, which were not possible with the common medical treatments and regular doctor visits. Another alumnus of this school is the home pregnancy test, which is based on lateral flow detection method that has affected the related biomedical sector significantly [9]. Down the road, the fast development of microfabrication methods helped to fabricate a wide range of nano/micro microelectromechanical systems (NEMS/MEMS)-based sensors including microfluidic chips, potentiometric biosensor based on various nanomaterials (e.g., ion-sensitive field-effect transistors (ISFETs), complementary metal-oxide-semiconductor (CMOS)-based biosensors and other miniaturized microdevices dedicated to biosensing applications [10,11]).

Due to the superior sensing characteristics of field-effect transistors (FETs), they have attracted huge attention in the biosensing research community with the motivation to create disposable, low-cost, miniaturized PoC devices based on FET sensors for home diagnostic purposes. The superior technological features of these sensors have huge potential for miniaturization, parallelization, ultra-low response times and seamless capability to be integrated with CMOS technology [12,13,14]. Historically, ISFETs were firstly introduced in the early 1970s by a subtle variation in the metal-oxide-semiconductor FETs (MOSFETs), which was removing the metal layer on top of the oxide layer [15]. Initially, it was conceived that by removing the metal gate on top of the oxide layer in MOSFET, space would be dedicated to an electrolyte solution as the fluidic gate in which a reference electrode (RE) can manipulate the surface potential of the oxide layer by changing the voltage. 

Moving toward chemically/biologically gated ionic sensitive FETs (Chem/BioFET) has brought about many advantages that could not be spotted in other potentiometric techniques. Being significantly miniaturized, relying on a very simple operational mechanism integrable with CMOS and competitive final cost were amongst the central reasons for Chem/BioFET popularity. From the invention of ISFET in 1976 on, different structures of ISFETs have been introduced for different bio-analytes in solution, which resulted in various Chem/BioFET structures [16]. Generally, the electrolyte gate is common in all Chem/BioFET structures that contain the analytes of interest for detection in an ionic solution (NaCL, KCL, PBS, etc.) buffer. The existence of the analyte tn will be detected by concentration or activity of the target molecule or just probing the presence/quantity of biomolecule on the sensing channel. The induced charge on the sensor will change the surface potential that will cause a detectable electron current in the conductive channel of the FET sensor. After physical sensing of chemical activities on the surface, the signal processing schemes can be conducted on the output voltage/current in the circuit. Afterward, the signal will be sent to a mobile device remotely for the online and distant screening of patient health by a medical doctor [17].

Considering the enormous potentials of Chem/BioFET in rapid detection with a limit of detection (LoD) down to fM, incredible sensitivity in comparison to other biosensors, along with their lower cost (when are integrated with CMOS technology), we realize that these sensors can be considered as the potential platform for manufacturing PoC devices. These PoC devices could be designed to address the need for doing rapid at-home tests, especially at the time of future pandemics like COVID-19 [18]. Label-free detection of infectious diseases could be realized by immobilizing antibodies, nucleic acids, aptamers, enzymes, microorganisms or artificial biomaterials on the sensing channels [19]. Immobilization of these bio-species will help to detect a specific disease biomarker in the solution in a label-free fashion when the chemical reaction occurs, or the target molecule approaches the surface. Furthermore, ISFETs can also be used for screening some infectious agents and their genomes by detecting the ions released by DNA polymerase [20,21], or the bacterial cells infected by bacteriophages [22] as well as measuring pH variations due to cellular metabolism and proliferation [23].

In this review, we have studied recent Chem/BioFET sensors dedicated to the analysis of infectious diseases along with Chem/BioFET physics of operation and the readout circuits. Section 2 describes the sensing mechanism in Chem/BioFET sensors. The third section gives an overview of the approaches used for sensing surface modification and functionalization of Chem/BioFETs for the detection of infectious diseases. Some readout circuits are reviewed in the fourth section, which are potentially useful for measuring the output parameters of Chem/BioFETs. The main focus of this section is CMOS-based circuits, which can pave the way for on-chip measurements and the development of affordable and handheld PoC devices. This review has been prepared in a way to convey information for all aspects of Chem/BioFET, which could be useful for engineering and biologists to accelerate their innovation for the detection of infectious diseases. 

## 2. Sensing Mechanism and Different Structures of Chem/BioFETs

**Figure 1:**
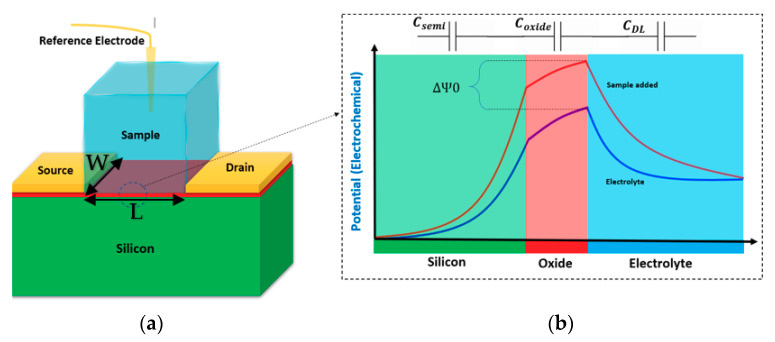
(**a**) The standard structure of a Bio-field-effect transistor (FET); (**b**) the corresponding potential diagram showing the effect of electrolyte on the potential of interfaces. The potential at the oxide layer and solution interface arises then will be decreased gradually to the solution potential. Adding the sample would manipulate this potential curve influencing the charge transport inside the silicon channel. There are three main capacitances involved in the system consisting of the oxide, channel depletion capacitance, and the solution double layer (*C*_DL_).

**Figure 2:**
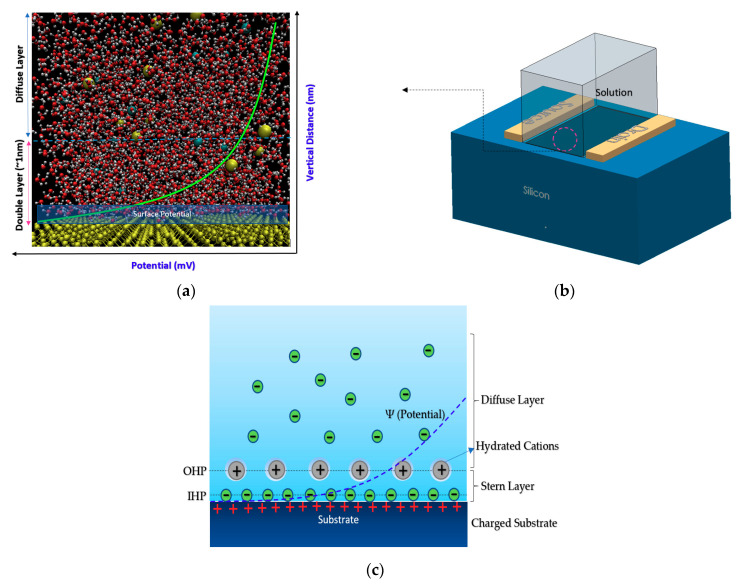
Showing how atomistic molecular dynamics simulation scales have been used to estimate the charge density on the surface of oxide layer (here SiO_2_): (**a**) Showing the silicon in atomic representation, which clearly shows the atomistic distribution of water and ions in the vicinity of surface silicon, which create a surface potential decaying into stable value in near zero in diffusion layer. The double layer has been shown whereby its effect would create a specific potential on the surface based on the ionic strength in the solution. Molecular dynamics can be used to estimate this potential in very complex solution containing biomolecules; (**b**) showing 3D sketch of a FET device; (**c**) potential and ions layers on the interface of electrolyte and substrate in sensor.

The principal physics behind almost all kinds of Chem/BioFET operation is the capability of these sensors to sense the charge effects on the surface through the induced electric field due to the existence of target molecules in the solution. When an electrolyte solution meets a solid surface (like silicon dioxide or any other oxides), a capacitive double layer (DL) is created, which consists of different layers such as a stern layer, inner Helmholtz (IHL), and outer Helmholtz (OHL) [24] (see Figure 1 and Figure 2 for better understanding). These layers encompass different concentrations of ions with respect to the vertical distance from the surface. This layered distribution of charges creates several ion layers that then can be modeled as capacitors on top of the surface of the oxide. Many attempts have been made to understand the interwoven effects of these layers and their contribution to the overall oxide-surface potentials [25]. The accumulated charge in the vicinity of the oxide surface (due to the creation of the electric double layer) changes the oxide electric field that eventually contributes to changing the potential at the outer surface of the sensing conductive channel. Afterward, the generated potential alters the space charge distribution inside the conductive channel and leads to source-drain current variations inside FET conduction channel. 

Unravelling the exact physical phenomena taking place at the semiconductor-oxide-electrolyte is of imperative importance when it comes to fabricating BioFET sensors more efficiently. Furthermore, this understanding becomes more important when we scale down to the nano BioFET structures, where the ions and molecular interactions dominate the electron conduction in the channel. This in-depth study would also enable us to decipher and control unexpected and intrinsic interfacial noises. Significant advancements have been made on this matter, which has considerably assisted the multi-scale design of ISFETs and the discovery of novel BioFET sensing materials. As the fabrication of these FETs progressed well, the development of theories to explain the physical phenomena was emerging in parallel to explain noises and some fabrication challenges [26,27]. The type of ISFETs on which a layer of the ion-sensitive membrane was in interaction with solution have been theorized perfectly. However, the direct interaction of electrolyte with oxide layer was not developed at the same pace [28]. 

As it is shown in Figure 1, due to the interaction of ions and the oxide layer, the double-layer capacitance will be created (*C*_DL_) on the proximity of oxide. The oxide layer capacitance, which is a built-in characteristic of this layer, will appear on top of the channel. The channel itself due to the depletion effect of the adsorbed charges on the surface will create another capacitor that will be modulated by the surface charge density and the electron/holes transport in the channel. Charge effects on the surface of the oxide layer would result in the source-drain current, which can be mathematically expressed as follows (we encourage readers to study an in-depth analysis in [28]):

(1) $$I_{D}=\mu C_{\mathrm{ox}}\frac{W}{L}\left\{\left[V_{\mathrm{GS}}-V_{t\left(\mathrm{ISFET}\right)}\right]V_{\mathrm{DS}}-\frac{1}{2}V_{\mathrm{DS}}{}^{2}\right\},$$

Equation (1) shows the current in the channel when the sensor is exposed to an electrolyte with varying potential and works in the linear operational region (*V*_DS_ < *V*_GS_ − *V*_t(ISFET)_). In this equation, the parameters are as follow: *µ* stands for average mobility in the channel; *W* and *L* are the width and the length of the gate, respectively; *V*_DS_ and *V*_GS_ are the drain-source voltage and the gate-source voltage, respectively; *C*_ox_ identifies the capacitance of the gate oxide. *V*_t(ISFET)_ is the threshold voltage of the ISFET, which can be expressed as follows:

(2) $$V_{t\left(\mathrm{ISFET}\right)}=E_{\mathrm{ref}}-\Psi_{0}+\chi^{\mathrm{sol}}-\frac{\phi_{\mathrm{Si}}}{q}-\frac{Q_{\mathrm{ox}}+Q_{\mathrm{ss}}}{C_{\mathrm{ox}}}-\frac{Q_{B}}{C_{\mathrm{ox}}}+2\phi_{f},$$

where *E*_ref_ identifies the potential of the reference electrode; $\Psi_{0}$ is the insulator-electrolyte potential; $\chi^{\mathrm{sol}}$ stands for the surface dipole potential of the solution; $\phi_{\mathrm{Si}}$ is silicon electron work function; *q* denotes the elementary charge; *Q*_ox_, *Q*_ss_ and *Q*_B_ are the charges in the oxide, charges in surface states and interface states and the depletion charge, respectively; and $\phi_{f}$ is the potential difference between the Fermi levels of doped and intrinsic silicon. 

All of the parameters in Equations (1) and (2) are constant values except $\Psi_{0}$, which shows the effect of surface potential on the oxide layer and affects *V*_t(BioFET)_. This will be changed by the ionic solution and biomolecular content of the solution that directly influences the current in the channel. Through mathematical modelling and using site-bonding theory (please read [29], and [30] for detailed information) the potential of the surface could be related to the pH of the bulk solution. The corresponding equations for more insight are as follow:

(3) $$\frac{\partial \Psi_{0}}{\partial pH_{s}}=\frac{\partial \Psi_{0}}{\partial \sigma_{0}}\frac{\partial \sigma_{0}}{\partial pH_{s}}=\frac{-q\beta_{\mathrm{int}}}{C_{\mathrm{dif}}},$$

(4) $$\frac{\partial \Psi_{0}}{\partial pH_{s}}=-2.3\frac{KT}{q}\alpha ,\mathrm{with}\;\alpha =\frac{1}{\left(\frac{2.3kTC_{\mathrm{dif}}}{q^{2}\beta_{\mathrm{int}}}\right)+1}$$

where α shows a dimensionless sensitivity parameter. The value of α varies between 0 and 1, which highly depends on the intrinsic buffer capacity and the differential capacitance. *K* is Boltzman constant, and *T* is the absolute temperature. Furthermore, $\sigma_{0}$ stands for the surface charge (charge on oxide surface and in general the interface between surface and electrolyte), *β*_int_ is the ionic capacity and *C*_dif_ denotes the differential capacitance.

The surface potential will be changed when a small variation takes place in the electrolyte ion concentration that could be the result of either a chemical reaction in a biological sample or simply the existence of charged molecules in the solution. Generally, adding a specific concentration of an analyte varies the pH of the solution, which can be correlated to the potential of the surface through Equation (4). This potential affects the source-drain current in the conduction channel. So, the threshold voltage of an ISFET is [31]:

(5) $$V_{t\left(\mathrm{ISFET}\right)}=V_{t\left(\mathrm{MOS}\right)}+\left(\gamma +2.3\alpha V_{\mathrm{th}}pH\right),$$

where *γ* is a pH-independent grouping of chemical potentials, *V*_th_ = *kT*/*q* denotes thermal voltage, and *V*_t(MOS)_ is the threshold voltage of a conventional MOSFET. Different operational regions of the ISFETs can be used for sensing including (1) Triode (or linear region), (2) strong inversion (or saturation), (3) weak inversion (or subthreshold) and (4) velocity saturation. As aforementioned, Equation (1) describes the drain current in the triode region. Drain current of a saturated ISFET (*V*_DS_ > *V*_GS_ − *V*_t(ISFET)_) is obtained by:

(6) $$I_{D}=\mu C_{\mathrm{ox}}\frac{W}{L}{\left(V_{\mathrm{gs}}-V_{t\left(\mathrm{ISFET}\right)}\right)}^{2}\left(1+\lambda V_{\mathrm{ds}}\right),$$

where *λ* is the channel length modulation factor. If *I*_D_ and *V*_DS_ both are held constant, *V*_GS_ will adjust to compensate for any changes in *V*_t(ISFET)_.

By assuming a zero bulk-source voltage and *V*_DS_ > 4*V*_th_, the operation of an ISFET in weak inversion region can be described by [31]:

(7) $$I_{D}=I_{0}\frac{W}{L}exp\left(\frac{V_{\mathrm{GS}}-V_{t\left(\mathrm{ISFET}\right)}}{nV_{\mathrm{th}}}\right),$$

where *I*_0_ is a positive constant current and *n* stands for the subthreshold slope parameter. 

In the velocity saturation region, the operation of the device can be expressed by [32]:

(8) $$I_{D}=v_{\mathrm{sat}}C_{\mathrm{ox}}W\left(V_{\mathrm{GS}}-V_{t\left(\mathrm{ISFET}\right)}-V_{D,\mathrm{sat}}\right),$$

where *v*_sat_ and *V*_D,sat_, respectively, stand for the saturated carrier velocity and the point when the drain current saturates.

These principles work for semiconductor-oxide-electrolyte Chem/BioFET regardless of their geometries and channel-gates arrangement. However, the principle might not be applied to some specific Chem/BioFETs, which are working based on the sensing properties of nanomaterials like graphene or carbon nanotubes (CNTs). This fact stems from the fact that the ion complexation on these surfaces might not follow the site-bonding theory. Mathematical modeling of the molecular interaction at the surface (oxide or nitride) is extremely difficult, thus prediction of surface potential will be impossible like what site-bonding theory provides. However, in terms of modeling the interaction in BioFET surface complex systems, recently atomistic molecular dynamics simulation has been recruited to analyze the ion complexation on the surface and measure the surface potential on the channel [33,34,35]. State-of-the-art molecular analysis has empowered researchers to get insight into the molecular interactions at the channel surface and estimate the double-layer capacitance considering the interaction of nanomaterials, oxide, ionic solution and proteins on the surface, which is not practical possible with current models. Figure 2a–c represents the surface potential in a very complex system containing ionic solutions.

In spite of the progresses in surface potential calculation modeling methods, there are still uncertainties in predicting the exact potential on the oxide surface when different “biomolecules” are available that do not affect the pH or ion concentration variations. Although the pH sensing mechanism is based on the detection of [H^+^] concentration, with surface modification, we can sense other ions in the solution that introduce other mechanisms of detection that are solely based on variation of specific ion concentration (e.g., [Na^+^] or [K^+^]) and no longer depend on [H^+^] [36]. These chemical FETs could be realized by immobilizing a sensing layer, which is only permeable to one or multiple ions in the solution. Knowing the dependence of sensor response on the concentration of specific ion in the solution (other than [H^+^]), we can design a wide range of ChemFETs capable of sensing existence of a biomolecule or chemicals that do not change pH, but the concentration of other ions. Enzymatic reactions can also be another mechanism of detection by which the analyte of interest can be detected by immobilizing surface with specific enzyme that can only react with the targeted protein in the solution. The enzymatic retractions contribute to creation of acidic molecules, which directly affect the pH of solution that can be detected by BioFET sensor [37]. In recent years, oligonucleotides have been introduced and gained a growing popularity in scientific community due to their contribution in direct detection of biomolecules in BioFET. The direct detection mechanism includes functionalization of oligonucleotides on the surface of sensor, which helps to screen the macromolecules in the Debye length on the surface, since they are much smaller than proteins (target molecules) [38]. Generally, the mechanism of detection in BioFET embraces pH variation, reaction-origin detection (e.g., enzymatic or redox), direct sensing of molecule based on Debye length screening and indirect methods such as oligonucleotides functionalization of surface to detect the DNA on the surface. There is still many in-depth studies required to unravel the complex surface phenomena at the interface of electrolyte, oxide and biomolecules. 

### 2.1. Chem/BioFETs Device Structures 

**Figure 3:**
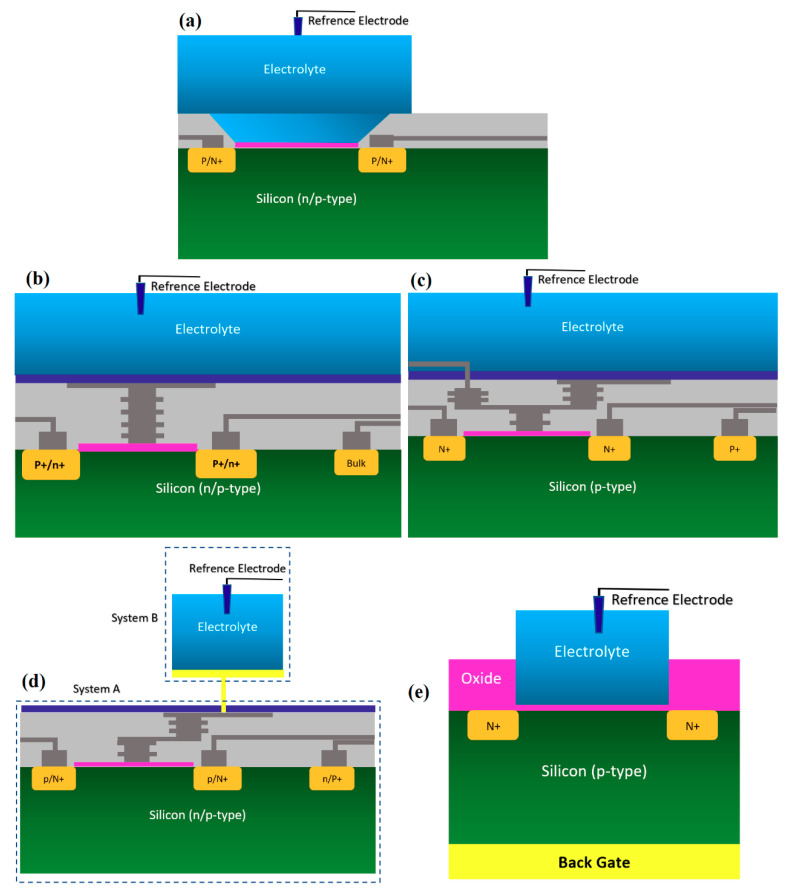
All structures of metal-oxide-semiconductor (MOS)-based ion-sensitive field-effect transistors (ISFETs) platforms that have been used for Chem/BioFET applications: (**a**) Representation of Oxide-electrolyte gate Chem/BioFETs, which include a reference in a solution on top of the oxide layer; (**b**) showing the floating gate structure by which the solution area has been separated from the conduction channel by an internal connection from the oxide layer on top of the channel to the solution and sensing membrane; (**c**) a demonstration of the integration of a floating gate with control gate, which helps to have more accurate control over the gate operation; (**d**) shows extended gate structure that allows us to have a bigger sensing area for biosensing purposes. Moreover, an extended gate creates enough room for further integration with the microfluidic system, which in most cases was not applicable to add them to small ISFETs; (**e**) oxide-electrolyte gate Chem/BioFET with the back gate in which used the back gate in order to manipulate the working point of the sensor by changing the depletion effects in the conductive channel.

**Figure 4:**
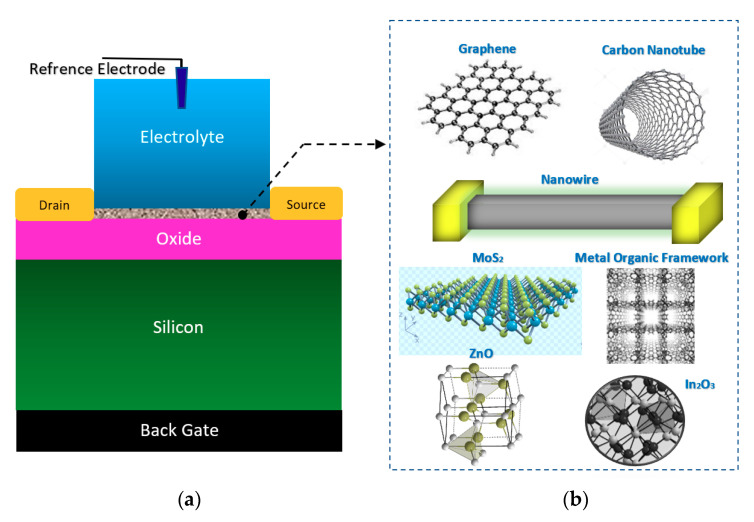
The general form of electrolyte and back gated structures of FETs, which have been used for the sensing elements in Chem/BioFET: This nanomaterial-based structure has been widely used for cell, DNA, enzymatic reaction and chemical sensing; (**a**) sensor structure; (**b**) the most frequent nanomaterials used as sensing element and main conductive channel.

BioFET sensors refer to all family of FETs dedicated to measure charge-induced field effects in different settings of bio-interfaces such as Gene-FETs (DNA-based FETs), Enzyme-FETs (Enzyme reaction detector) and Cell-FETs (FETs with biological cells as their gate). While the biological targets in these sensors are different, the mechanism of their operation is based on ISFETs operation. The invention of ISFETs goes back to 1972 when Piet Bergveld introduced the technology and tested it for pH measurements of NaCl solution. From Piet’s ISFET invention in 1972 forward (about 50 years of research works), many geometries of ISFET sensors have been developed that generally can be categorized into six main groups based on their gate operation. These structures are conventional oxide-electrolyte structure (only metal of MOS is removed, and an oxide layer is deposited), unmodified CMOS technology, floating gate, extended gate, double gate structures and top gated structures, which can be modified with different nanostructures and reinforced with materials (See Figure 3). These structures specifically or in a combination with other forms have been recognized as the basis for further development of other biological FET sensors such as graphene-FETs (GFETs), CNT-FETs, nanowire FETs (NW-FETs) or other novel sensing materials such as MoS2 and Metal-Organic Frameworks (MOFs) [39,40,41,42,43] (See Figure 4). 

#### 2.1.1. Oxide-Electrolyte Gate Chem/BioFETs 

**Figure 5:**
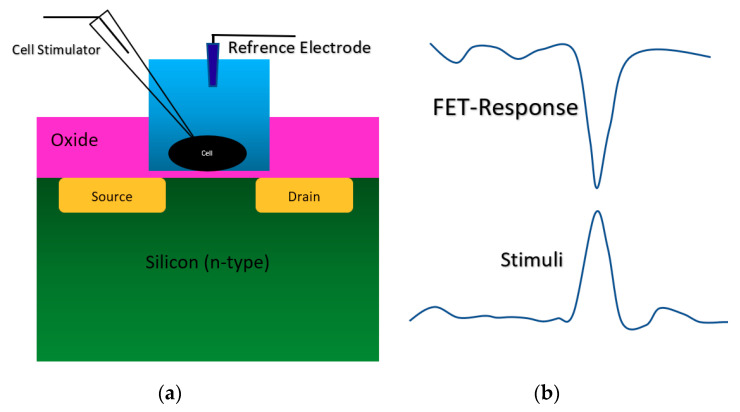
The stimulation and the response of FET sensor from a cell on top of an oxide-semiconductor. (**a**) Sensor structure for culturing cell on the oxide layer on top of the conductive channel; (**b**) the stimuli and the sensor response at the same time, which shows the successful loading and recoding a pulse onto/from the cell using ISFET sensor. The FET-response is “current”, and the stimulus is voltage.

According to Figure 3a, this category of Chem/BioFET structures has been the same as the early ISFET design. From when it was invented, it has been the most applied Chem/BioFET structure in literature [44] for pH and biological analysis. The metal on the MOS sensors (as the gate) was removed and an oxide layer such as SiO_2_, Al_2_O_3_ or Ta_2_O_5_ was deposited on the opened gate area on top of the conductive channel, which could then a layer of nitride be deposited although it is not necessary. This sensing platform has come with different arrangements, but the only difference with the type of sensing mechanism (shown in Figure 3) is that the conductive channel here is the silicon itself, and no other materials are used for the enhancement of sensing capabilities. For these sensors (see Figure 3) SiO_2_ and Si_3_N_4_ have mostly been used as the oxide and nitrite layer, respectively, which play the role of dielectric. Generally, Si3N4 has been deposited on SiO_2_ to control the desired dielectric value in sensor. While the deposition of these oxides is completely well established, there are some concerns regarding the usage of these materials for ions sensing, since the oxide sites on the outer surface create a huge number of charge-trapping sites that will not be removed easily and will contribute to unwanted parasitic responses from the sensor [45]. The FET developed in [40] recorded a response against different concentrations of *Pf*GDH protein spiked in buffer and serum with corresponding calibration of source-drain current versus gate voltage for different concentration of Plasmodium falciparum glutamate dehydrogenase. When concentration changed from 100 fM to 10 nM the sensor response changes (current value) changed from 0.5 µA to 0.8 µA in *V*_gs_ = 0.88 V. This structure was initially used for detection of Na^+^ and H^+^ ions activity for monitoring of extracellular ion pulses measured with a guinea, pig taenia *coli* [46]. In their design, the p-type silicon channel was brought in contact with SiO_2_ layer as the insulator for interaction with electrolyte. Standard oxide-electrolyte gate structure was used for evaluation of the bacterial deposition, which accumulates under conditions normally employed for telemetric monitoring of changes in human dental plaque pH [47]. As an early attempt for neuronal recording, a neuron was mounted on a thin insulating layer of a gate oxide on n-type Si in an electrolyte like the one shown in Figure 5a in which a positive bias voltage was applied to the silicon to deliver an accumulation of moveable, positive defect electrons near the surface (strong inversion). 

As it is demonstrated in Figure 5b, when a positive voltage happens in the neurons during a voltage stimulation, the surface potential of silicon in the conduction channel region will be increased, which causes a reduced current in the channel (the curve shows current response). The neuron cells stimulation and recording were perfectly recorded by the oxide-electrolyte gated. This structure was used for different physicochemical settings such as immunodetection of anaerobi bacteria, which has been developed using *Clostridium thermocellum* cells [48], adhesion analysis of a single neuron cell on oxidized silicon [49], analysis of hybridization of synthetic homo-oligomer DNA sequences [50], electrogenic cell monitoring [51], cellular metabolism monitoring [52], monitoring excitable neurons of rat brain [53], cardiac muscle [54], sensor and fluidic packaging for cellular monitoring [55], cell proton transport mechanism analysis [56]. This structure has been recruited for cellular activity analysis with focusing on local pH measurement near the surface [57,58], and the adhesion analysis of cells on the substrate by measuring the pH of cells far from their culture area [59]. In the most recent couple of years, an ISFET sensor with oxide-electrolyte structure has been used for cell analysis and pH measurements. A SiO_2_-Ta_2_O_5_ oxide gated ISFET was used for live-cell monitoring by measuring the pH variations around cells on the gate [60,61]. In another attempt, Si_3_N_4_/Ta_2_O_5_ oxide gated ISFET was used for studying the self-assembly of photosynthetic proteins [62].

#### 2.1.2. Chem/BioFET Based on Standard CMOS

**Figure 6:**
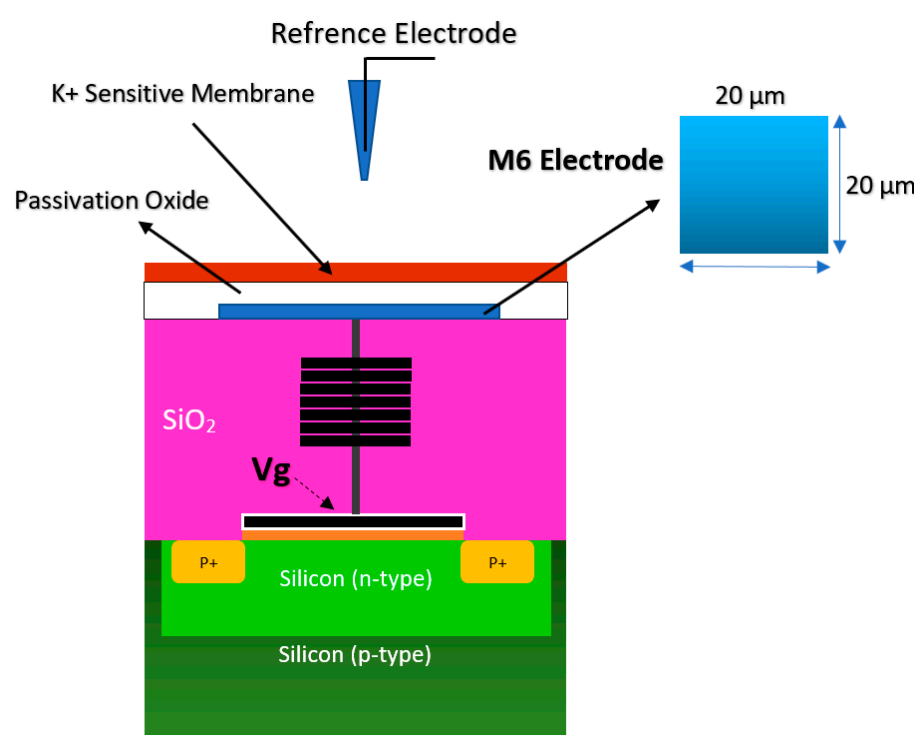
The standard complementary metal-oxide-semiconductor (CMOS) ISFET for rapid detection of *E. coli* bacteria using an unchanged CMOS structure coated with potassium sensitive membrane.

**Figure 7:**
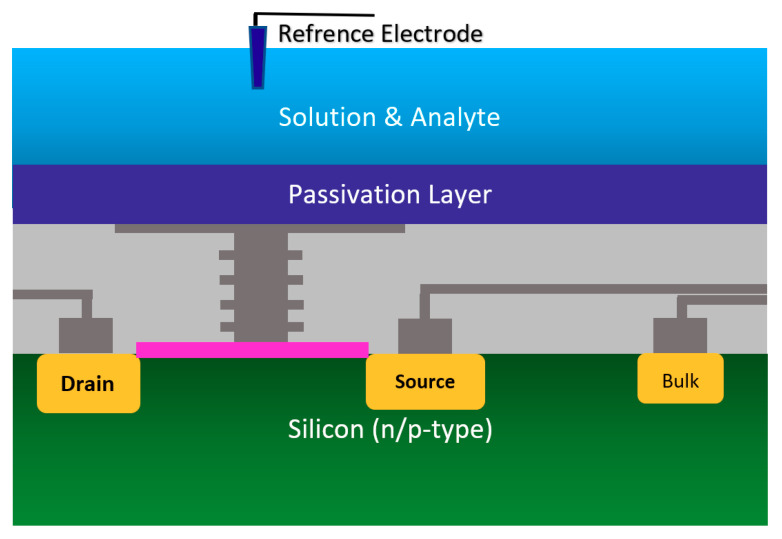
The standard CMOS ISFET for cellular monitoring application.

Using the well-matured CMOS technology as the main platform for the development of ISFET was a smart step toward the development of very standard ISFET sensors for biological applications [22,63]. As an example of the transfer characteristics, the fabricated circuit delivers a linear dynamic range of 2.5 V that allows each individual ISFET to operate as a pH sensor in the array [58]. The ISFETs have a threshold voltage of −1.5 V and a sensitivity of 46 mV/pH [58]. Using unchanged CMOS structures (see Figure 3b) for ISFET sensors comes with some advantages that make this structure very competitive in comparison to other Chem/BioFET structures. CMOS adaptation makes Chem/BioFET-based sensors very scalable and low-powered, and are being considered as two main demanding characteristics for handheld PoC devices. As shown in Figure 6, this sensor is fabricated by connecting the elongated gate of a transistor up to the solution that perfectly separates the solution from the sensor area. The gates, through some metal connectors buried in the oxide layer of CMOS, will be connected to the layers of Si_3_N_4_, SiO_2_, etc., which play the role of ion complexation surface for H^+^ sensing [22]. As an example of the unchanged CMOS sensor, Milgrw et al. [63], used a 16 × 16 array of these sensors for direct extracellular imaging. The structure of this sensor has been shown in Figure 7.

Along this line, new CMOS-based inventions appeared for chemical sensing. A needle-like ISFET sensor was developed for probing very small spaces in biological and chemical samples with the sensitivity of 45 mV/pH [64]. A commercial 0.25 um CMOS technology was modified as a standard ISFET for pH measurement by Georgiou and Toumazou [65]. The dimensional and shape analysis has been studied by Sohbati et al., for taking into account all the important geometrical parameters playing a part in the operation of ISFET based standard CMOS technology [66]. A common feature in the ISFETs based on the CMOS technology is the use of SiO_2_ and Si_3_N_4_, or two of them, which create additional capacitance above the silicon channel that affects the ISFET sensitivity (and also the parasitic errors). The coverage of all parts of the sensor with this thick oxide/nitride layer is a disadvantage of the sensor, which results in an unwanted capacitance that affects the threshold voltage of the sensor as the ion complexation/trapping sites on the oxide/nitrite surface will influence the electron current in silicon for next tries [65,66]. This technology was reflected in successful real applications, working based on pH measurement such as detection of nucleic acid amplification [67] and next-generation genome sequencing [68]. 

#### 2.1.3. Floating Gate Chem/BioFET 

**Figure 8:**
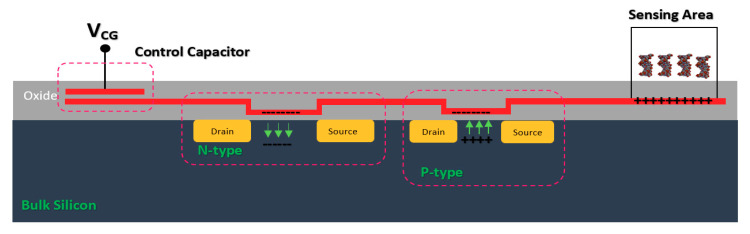
The floating gate structure and a real chip designed by Barbaro et al. for Label-free DNA analysis. This sensor contains 80 biosensors that are placed in 2 channels. Additionally, on this chip, a microfluidic system has been bonded which provides access to the sensing areas for test solutions and reagents.

Commonly, a floating electrode is used to protect the oxide sensing region on top of the channel and also provide better control over the sensitivity of the device in different operation settings, which is a preferred condition when it comes to having uniform modulation of the sensor. As shown in Figure 8, the floating gate-based Chem/BioFET structure includes two gates, one for exposure to biological samples and another one for controlling the gate. This gate structure has been used successfully for the investigation of DNA charges on the surface of oxide as depicted in Figure 8. 

The sensor illustrated in Figure 8 operates without an RE as the floating gate was used for modulation and control of the gate on oxide in which the threshold voltage varied between −0.8 to 0.8 Volts in different stages of DNA sensing procedures [69]. The floating gate structure has been used in another Chem/BioFET sensor for oxide functionalization of conductive organic electronic [70]. In [70], the sensor has also been tested without an RE as the tests were performed in dry conditions. In an interesting job, the floating gate structure also was successfully tested for the realization of programmable ISFETs and used that for DNA analysis [71,72]. In terms of investigation of the neuron interactions, floating gate structure was used as a sensing platform by Cohen et al. [73]. They designed a floating gate structure that provides a wide gate area for monitoring the chemical reactions, which makes it a good sensor for integration with microfluidic [73]. Chemoreceptive neuron MOS (CνMOS) transistors have been used for electrochemical recordings of exocytosis from populations of the mast and chromaffin cells [74]. This sensor arrangement by means of a floating gate structure (coexistence of control gate, sensing gate and floating gates together), allows the simultaneous control of the electrolyte and ions quiescent point to be independently controlled. The sensor is also CMOS-compatible and physically isolates the transistor channel from the electrolyte for stable long-term recordings [74].

#### 2.1.4. Extended Gate Chem/BioFET 

**Figure 9:**
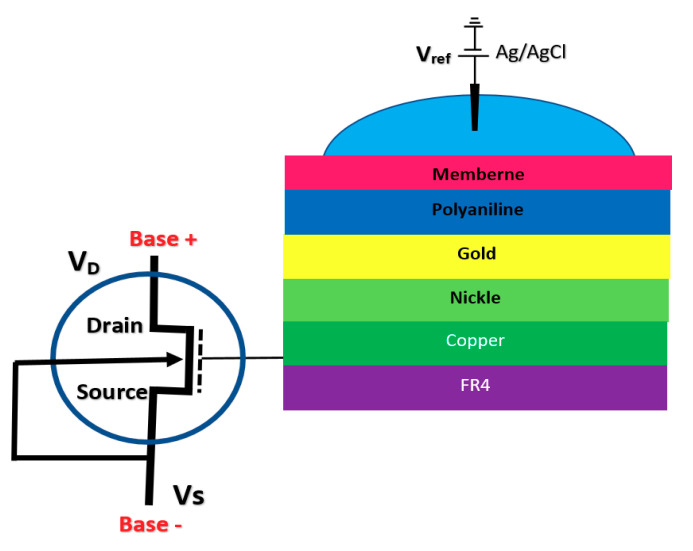
Extended gate sensor developed by Kaisti et al., which shows a handheld system for biochemical analysis utilizing a transistor and a specifically designed gate to transfer the detection unite to the sensing FET.

The integration of FET sensors with a microfluidic platform for directing the solution and biomaterials toward the sensor is a big challenge in biosensor integration. However, a good approach for alleviating this issue is to extend the gate of the FET sensor compact area to meet the biological matters [75,76]. This structure helps to have a much simpler fabrication process, as the sensing area can be fabricated according to the design demand of the fluidic section. Regular FET biosensor designs as discussed in previous sections (oxide-electrode gate and floating gates) come with some challenges mostly associated with the inevitable surface chemistry of ion complexations and the following ion trapping phenomena, which leads to the development of noise in the output signal [75]. Extended gate Chem/BioFETs have been designed to improve these deficiencies, which are investigated in literature with different applications for pH measurement of ion solutions and other bio-interfaces [16]. In comparison with ISFETs, extended gate FETs have shown much better stability in terms of chemical, thermal and incident light disturbances in parallel with greater sensitivity [16,77]. Figure 9 demonstrates an example of an extended gate FET sensor that has been used as a platform for biochemical analysis [76]

**Figure 10:**
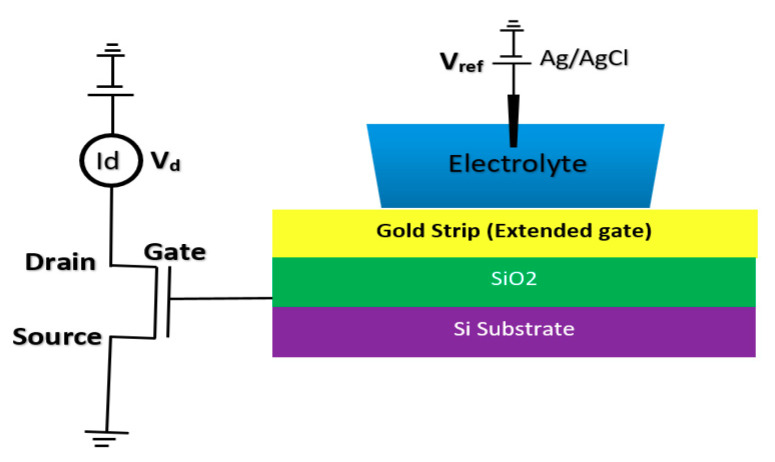
Extended gate Chem/BioFET structure in which the gold gate was deposited on SiO_2_ and Si substrate. The extended gate was connected to the fluidic channel through a liquid cell on top of the sensor.

Due to the specific characteristics of extended gate FET for biosensing applications, many are designed for various detection applications such as pH, urea, glucose, calcium ion, DNA, and immunosensors, which have been registered. A complete review of these sensors is done in other work by Pullano et al. [75]. An extended gate sensor was used for direct potentiometric serological diagnosis toward detection of Bovine Herpes Virus-1 (BHV-1) pathogen by Tarasov et al. [77]. Their design has been shown in Figure 10, which demonstrates an extended gate connected to the gate of a MOSFET. In their experiment, concentrations of p53wt changed from 50 pM to 10 nM, whcih resulted in Ids jumps ranging from about −5.0 × 10^−8^ A up to about −5.0 × 10^−7^ A.

#### 2.1.5. Double Gate Chem/BioFET 

**Figure 11:**
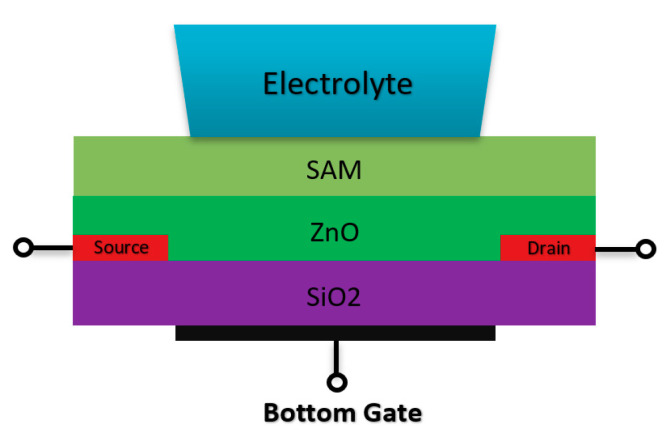
Double gate structure Chem/BioFET designed based on the double gate structure. The SiO_2_ is placed at the back of the channel (ZnO) and the electrolyte solution has been placed on top of the sensing layer on top of the conductive channel.

Double gate sensors have the same structures, which are presented before in terms of the drain/source and channel operation mechanism. These sensors mostly operate under an RE while another gate at the back of the sensor helps to manipulate the sensors’ operation point. Due to this further operation control using back gate, they were intended to go beyond the Nernstian limit, which defines the ISFETs sensitivity limit [78,79,80]. Spijkman et al., in a very interesting piece of research work, introduced a new configuration of ISFETs using double gate concept in which they could push the Nernstian limit to about 2.25 V/pH using a self-assembly polymer as the solution interface gate on top of ZnO channel with a back gate that contributes to the creation of bottom capacitance [79,80] (Figure 11 shows the double gate sensor). 

**Figure 12:**
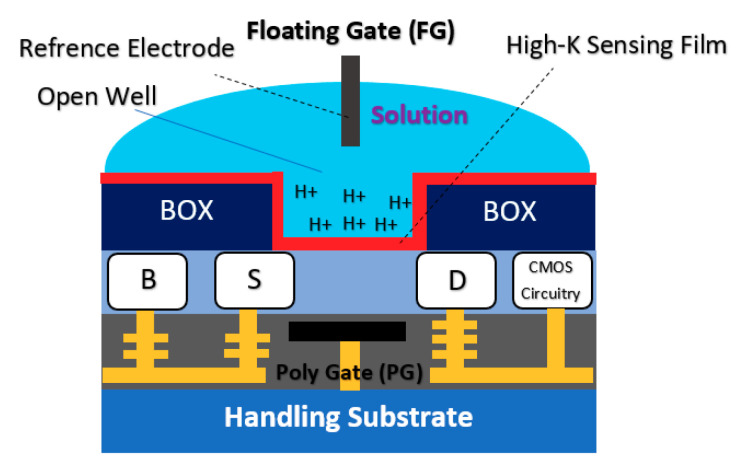
Dual-gate ISFET sensor structure developed in a standard 0.18 µm SOI-CMOS process and through a backside process the back gate has been developed to make a double-gate structure. The sensor completely is working based on the CMOS readout.

The double gate structure was also reported by Huang et al., as an efficient way to improve the performance of standard CMOS-based ISFETs. They reported a dual-gate ISFET sensor structure, as depicted in Figure 12, which is developed in a standard 0.18 µm SOI-CMOS process followed by an additional backside process [81]. Using poly gate (PG) (see Figure 12) as the second gate of double-gated CMOS standard ISFET structure, helped them to significantly improve the characteristics of ISFETs such as 155 times improvement in signal-to-noise ratio (SNR), 53 times improvement in drift rates, 3.7 times hysteresis reduction, and last but not least, 7.5 times sensitivity increment [82]. Back gate structure has been used frequently in FET structures in which nanomaterials (please see Figure 3b) were deposited on the oxide and played the role of main conductive channel. The most noteworthy ones are CNT-based FETs [83], grapheme-FETs [82], silicon nanowire (SiNW)-FETs [84], MoS2 FETs [85], MOF FETs [42], ZnO FETs [42,86] and other nanomaterials utilized the double gate and in the form of a solution gate and a back gate for sensing procedures.

### 2.2. Chem/BioFET Structures Used for Infectious Disease Screening

As previous discussions have shown, various structures of ISFETs have been employed in Chem/BioFET biosensing systems. These sensors have also been frequently used for the detection of infectious diseases. Almost all of the Chem/BioFETs used for infectious diseases have been based on the electrolyte gate on the main channel. However, there were some examples of other structures as well.

**Figure 13:**
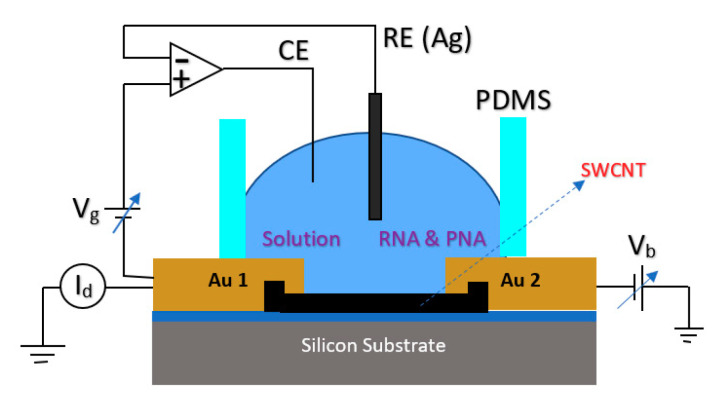
FET structure used for detection of RNA. In the figure, Au 1 and Au 2 demonstrate the source and drain pads, respectively. The gate potential has been applied through the Ag/AgCl as RE, and in this picture, the *V*_b_ is the bias voltage of the source-drain.

For detection of a sequence of Hepatitis C Virus (HCV) at very low concentrations down to the pM range, a Peptide Nucleic Acid (PNA) CNT-based FET structure sensor was used in which the sensor was working based on the Ag/AgCl solution gate in the electrolyte [87]. The structure of the fabricated sensor is shown in Figure 13, which consisted of metallic SWNTs that would lower the sensitivity of the devices for sensing applications. In this work, to lessen the effect of contact residence between single wall CNTs (SWCNTs) and the metal layer, the contact areas were passivated with microlithograpically patterned Al_2_O_3_ (50 nm) or self-assembled dodecanethiol (DDT) monolayers [87].

**Figure 14:**
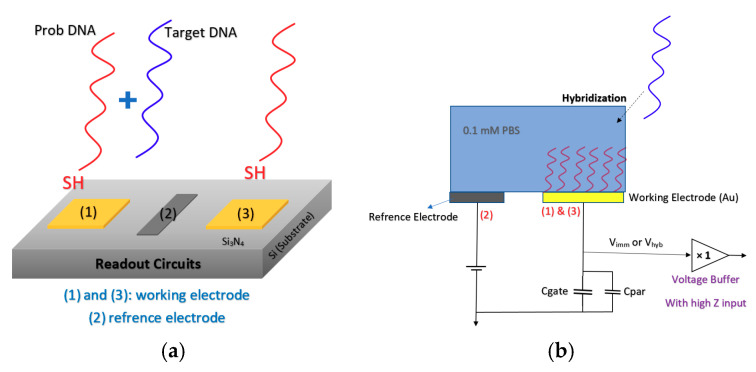
The sensor proposed by Lee et al. in order to detect the RNA of H5N1 AIV-based on hybridization detection on the surface of the gold electrode: (**a**) Showing the working electrode and reference electrode arrangement which are dedicated for probing and reference reaction; (**b**) a brief schematic of circuit and the way it has been connected to the reference and working electrodes.

Lee et al. [88] developed an extended gate Chem/BioFET structure (see Figure 14) that has been manufactured based on the 0.35 µm CMOS process, which was integrated with working electrodes, an RE and readout circuits into one package. A detectable range of 88.3 dB and an LoD of 36 µV were reported for this sensor, which led to a successful Chem/BioFET for the detection of oligonucleotide sequences derived from the H5N1 avian influenza virus (AIV). The structure of this sensor is introduced in Figure 14a,b, which shows a conceptual view of the integration and working principle of the device. All of the electrodes were built in the device area, which included working electrodes, an Au (numbers (1) and (3)) modified along with DNA strands and an RE (Al, number (2)) that determines the potential of the analyte solution. After the introduction of the sample on the functionalized extended gate, the signal will be processed in the CMOS circuitry.

**Figure 15:**
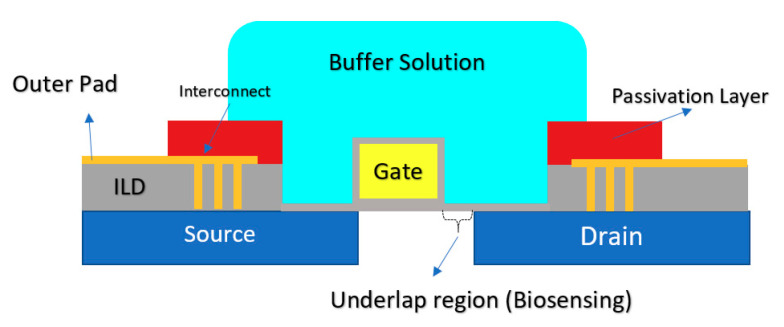
A CMOS-based Chem/BioFET structure using an underlap-FET biosensor: The cross-sectional of a water droplet is shown as the solution on top of the sensor. This sensor has been used to show the effect of wettability on the sensor characteristics and in particular sensitivity.

Another CMOS-based Chem/BioFET platform is shown in Figure 15 that utilizes a gated area on the main channel while the solution is introduced onto the sensor through an underlap region [89]. In the sensor shown in Figure 15, to examine the effect of hydrophilicity and hydrophobicity on the response of the sensor, a thin film of CYTOP^TM^ and silicon nitride has been used as the hydrophobic and hydrophilic passivation layers, respectively. The sensor finally was tested toward detection of surface antigen and its specific antibody of the AIV. This work introduced a method for enhancement of the sensitivity of CMOS-based Chem/BioFET sensors for better detection of infectious disease and in particular influenza [89].

**Figure 16:**
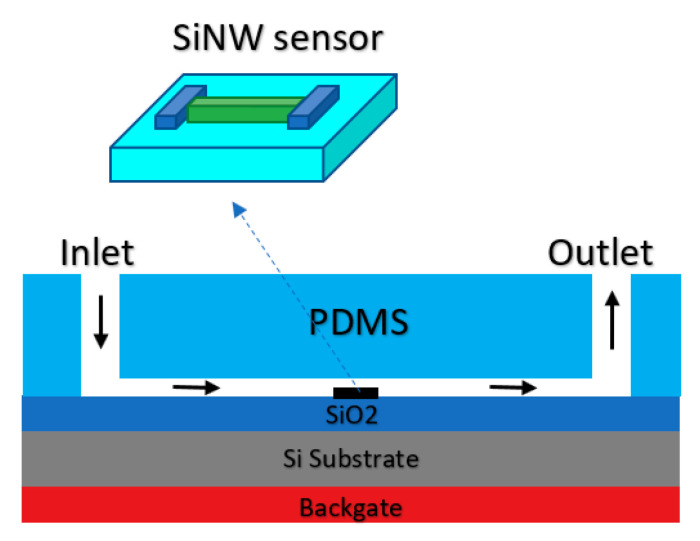
The schematic of silicon nanowire (SiNW) integrated with PDMS microfluidic channel which was designed to direct the flow of sample of interest toward the solution. An Ag/AgCl electrode is integrated by placing its tip in the inlet of the microfluidic channel.

As it was shown in Figure 2, many sensors have employed nanomaterials as the sensing element, which are considered as conductive channels. Almost all sorts of these sensors are working based on the back-gate structure. Therefore, double-gated Chem/BioFETs considering one of the gates as a RE in the electrolyte solution. One of these structures employing both the back gate and also a solution gate was implemented on the SiNW-based sensor for detection of H5N2 AIV in a very dilute solution [90]. To facilitate the integration of the RE with the SiNW, it was positioned inside the solution where the fluid was directed inside, as shown in Figure 16.

The ultrasensitive detection of H5N2 AIV was demonstrated using a reusable SiNW FET, which was made possible by the reversible surface functionalization on the SiNW via a disulphide linker. They reported a successful reversible surface functionalization, which was then examined by electrical and microfluorescence methods. By this innovation, they could reach detections of very dilute H5N2 AIV at 10^−12^–10^−17^ M [90].

**Figure 17:**
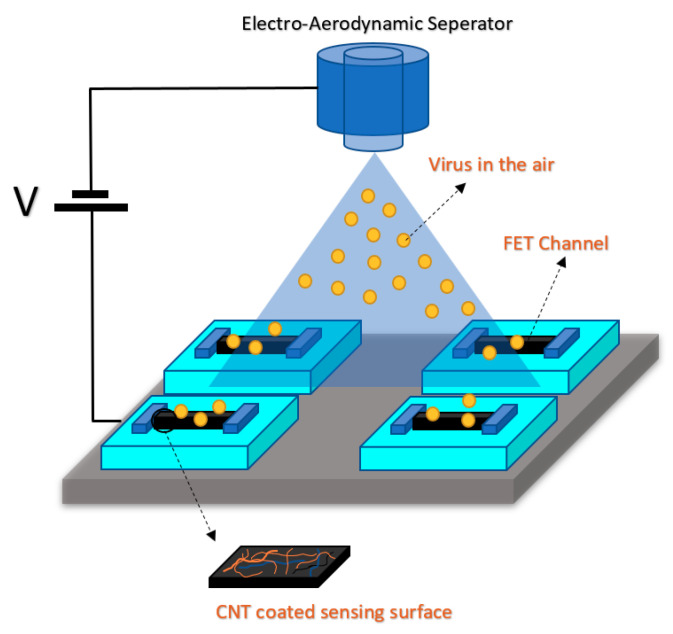
The carbon nanotubes (CNT) sensor working based on a back-gate biasing and the sample is introduced by an electro-aerodynamic separator without using wet samples.

As a back gated structure for SiNW sensors, based on a CMOS fabrication process, a label-free and specific DNA detector were devised in which by applying rolling circle amplification (RCA) on SiNW-FET, they could get higher sensitivity and better performance. Back gated structure was also used for direct detection of airborne viruses without using any solution and wet fluidic section [91]. According to this method, the virus–antibody connected particles are delivered to the FET during detection in which the regular pre-treatment and the antibody binding step on the FET channel are not required. In this method, they have eliminated the washing process for the virus–antibody binding [91]. On top of the sensor area, a layer of CNT was coated, which was used for the detection of virus deposition from the electro-aerodynamics separator (see Figure 17).

**Figure 18:**
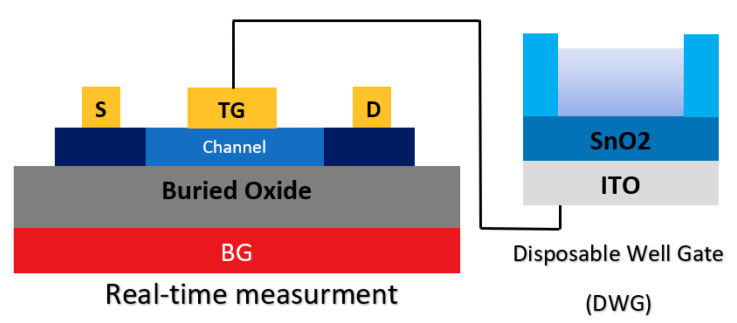
Illustration of the avian influenza virus (AIV) sensor configuration, which is a combination of double-gate and extended gate configuration.

In another study, the integration of a double gate and extended gate was studied to introduce a new sensor structure (see Figure 18). A so-called ‘disposable well gate’ was connected to the top gate of a channel, which was used to detect AIV [92]. This research reports a FET-based AIV sensor that was capable of detecting nucleoproteins within 30 min, down to an LoD of 10^3^ EID_50_ mL^−1^ from a live animal swab sample [92].

The back gate structure in another Chem/BioFET was used for Ebola virus disease (EVD) detection [93]. Reduced graphene oxide (rGO)-FET, which was controlled by a back gate was used for real-time detection of the Ebola virus antigen [93]. The same gating structure was used for developing an indium tin oxide (ITO) NW-based FET for DNA biosensing, which was used in particular for the detection of hepatitis B virus (HBV) based on ITONWs [94].

Based on our review, it seems there were a limited number of papers, which have used standard FET sensor fabricated based on CMOS technology for infectious disease detection. However, many of them have been fabricated based on laboratory microfabrication techniques. As far as the gating structure is concerned, many of Chem/BioFET infectious detector structures have been based on the top-gated type that generally is a solution gate with an RE. In the next chapter, we have reviewed the biological interface of the aforementioned Chem/BioFETs dedicated especially to infectious disease screening.

## 3. Surface Modification and Functionalization of Chem/BioFETs

The operation of FET biosensors depends on the bioreactions that occur on their surface [95]. Their efficiency and preciseness are contingent on the selectivity and availability of the bio-recognition elements (BREs). Therefore, proper surface functionalization is imperative for optimizing the BRE immobilization, enhancing the sensitivity, preventing unwanted reactions and minimizing the noise [96]. Additionally, the type of material used to cover the sensor’s surface to increase its biocompatibility and surface chemistry play a crucial role in improving the performance of the sensor [97]. As mentioned in the previous section, a wide range of substrates is utilized for this purpose including gold, nanowire (NWs), CNT, graphene, glycan, etc. [98]. The use of nanomaterials with extraordinary characteristics such as small size, high chemical and mechanical stability, considerable electrical conductivity, nontoxicity and high surface-to-volume ratio offers an optimum sensing area for operating an accurate detection [99]. Especially, one-dimensional nanostructures improve the sensor’s performance significantly and increase the LoD down to attomolar levels [100]. These structures enable label-free electrical detection of biospecies in a sensitive and precise manner [101]. Table 1 compares some of FET biosensors reported recently for detecting various infectious agents, especially different viruses. Herein, after introducing different materials useful for covering the sensors’ surface in Section 3.1, different type of BREs will be reviewed in Section 3.2.

### 3.1. Surface Materials

#### 3.1.1. Nanowires

**Figure 19:**
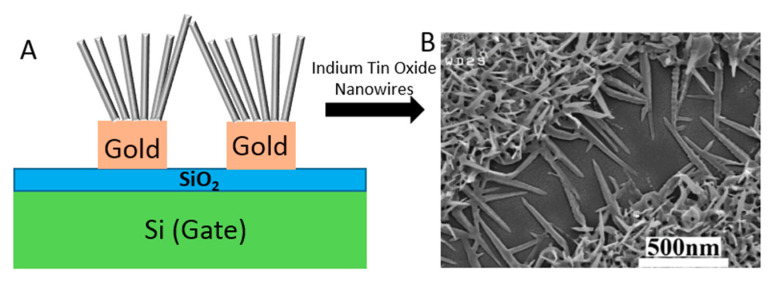
The configuration of ITONWs-FET genosensor. (**a**) Si/SiO_2_/Au/ITONWs device; (**b**) the FESEM images of indium tin oxide (ITO) samples.

NW-FETs are one of the most used categories of Chem/BioFETs [117]. In these systems, the channel and gate of a standard Chem/BioFET are functionalized with NWs and BREs [118]. Despite their different configuration, they operate through the same procedure. NWs can be fabricated through bottom-up (chemical etching, vapour-liquid-solid, and oxide-assisted growth) or top-down approaches from semiconductor materials [119]. SiNWs are among the commonly preferred alternatives because of their facile fabrication and modification process [120]. Their surface should be functionalized with specific BREs to be sensitive to the target viruses. This can be done through physical adsorption and chemical cross-linking [121]. After exposing the functionalized surface to the sample, an electric field is induced onto the NWs and changes their conductivity as a result of the interaction between the charged target and receptors [122]. Several types of biological interactions such as antibody–antigen, protein–ligand, and oligonucleotide hybridization can be inspected on the surface of NW-FET biosensors [123]. Recently, several studies have focused on developing novel biosensing platforms for detecting viruses. For example, a very recent study was conducted for detecting the influenza virus (IV) using a SiNW-FET-based biosensor, which was fabricated through CMOS technology. This system was able to spot down to 1 fM of the target protein [102]. As illustrated in Figure 19, another FET genosensor was designed to detect HBV based on ITONWs, which facilitated the surface functionalization and hybridization process significantly. After coating the nanowires with gold, they were modified with single-stranded DNA (ssDNA). This arrangement was successful enough to identify 1 fM of the viral genome in 37 s and discriminate the target from other oligonucleotides [94].

#### 3.1.2. Gold

**Figure 20:**
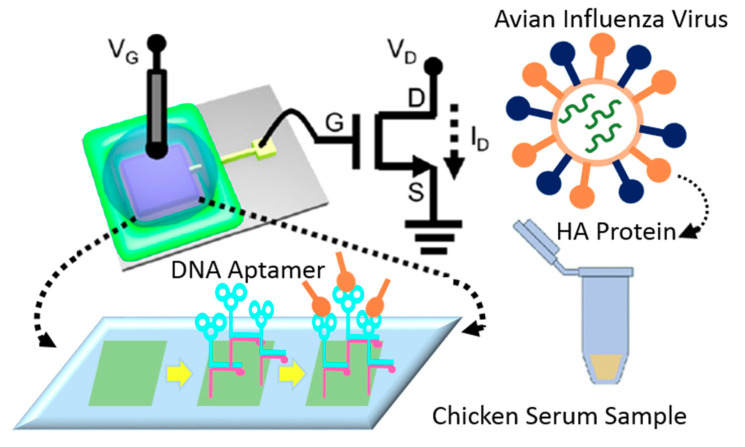
The illustration of the FET-based aptasensor for detecting AIV in chicken serum. The surface of the gold electrode was modified with specific DNA aptamers for identifying the surface protein of the virus. The interaction between the probe and the target resulted in the structural change of the aptamers which was recorded as the surface potential.

Au-modified surfaces represent a popular approach for surface functionalization of FET-based sensing systems since they provide a stable platform for immobilizing the BREs, boosting the electron shuttle rate, and upgrading the performance of the device [95,124]. Furthermore, the high biocompatibility of these surfaces preserves the functionality of the BREs and optimizes their efficiency in interacting with target biomolecules [125]. More importantly, the wide available area that is provided by gold nanoparticles enables the immobilization of a large number of receptors and accordingly augments the sensitivity of detection [126]. In recent years, several studies employed this strategy for designing biosensors for detecting viral particles. For instance, a reusable FET-based device was fabricated to identify AIV, using aptamer-modified gold microelectrodes (see Figure 20). The hemagglutinin (HA)-specific aptamers’ interaction with the target biomolecules alters the surface potential, which is recorded as a signal. The LoD was reported at 5.9 pM, which shows the preciseness of the detection in chicken serum. The easy and cheap fabrication process of this portable platform makes it an ideal approach for PoC diagnosis of viral particles in biological samples [104]. In another study, an immune-FET was designed for detecting the capsid protein of HIV. The gate of the device was covered by a monolayer of gold to provide a biocompatible area for immobilizing target-specific antibodies. The ultra-sensitive recognition power of this strategy (LoD = 30 × 10^−21^ M) enables reliable, economical and portable detection of single molecules [105] (see Table 1).

#### 3.1.3. Graphene

**Figure 21:**
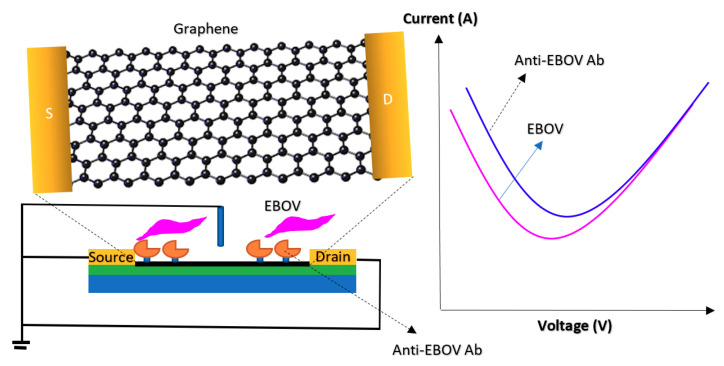
The illustration of the reduced graphene oxide (rGO)-FET immunosensor for Ebola Virus recognition and Surface modification and the conductance changes that occur upon attachment of the antigen to the antibody.

Graphene is the other widespread material for surface coating in biosensor designs [127]. It has several other forms such as graphene oxide (GO), rGO and graphene nanoribbon (GNR), which have been extensively used in the structure of sensing devices because of their chemical, mechanical, and electronic attributions [128,129]. This biologically friendly carbon-based material provides a wide area with numerous anchor spots for functionalizing the surface with diverse nanoparticles, polymers or any other signal amplifier before immobilizing the BREs. Additionally, it has exceptional electrical conductivity, high capacitance, low contact resistance and tunable ambipolar field-effect behaviors. Since it increases the electron transfer rate, the response time of the biosensor decreases significantly [130,131]. Besides, its cost-effectiveness, easy fabrication and biocompatibility turn it into one of the most preferred materials for coating the surface of virus-specific FET-based bioassays. As an instance, Jin et al. proposed a FET-based immunosensing device for the detection of the Ebola virus. As depicted in Figure 21, the surface of the sensor was functionalized by rGO before immobilizing antibodies. The LOD was reported 2.4 pg·mL^−1^ of the target glycoprotein in spiked serum samples [93].

In another study, a GFET system was reported for detecting IV and studying the antiviral medications’ effect on it. Sialic acid was used as a probe for recognizing the HA on the surface of the virus. Besides, the repressive effect of “zanamivir” on this interaction was investigated. This work represented the compatibility of these platforms for quantifying biological reactions in biosensing and drug development applications [113]. Aspermair and colleagues constructed a FET aptasensor for the identification of human papillomavirus (HPV). The surface of the sensor was characterized by rGO, pyrene and RNA aptamers to spot viral proteins in saliva samples. They successfully detected as low as 1.75 nM of the target molecules, which indicated the competency of the device in real-time detection of viral infections [109].

#### 3.1.4. Carbon Nanotubes

**Figure 22:**
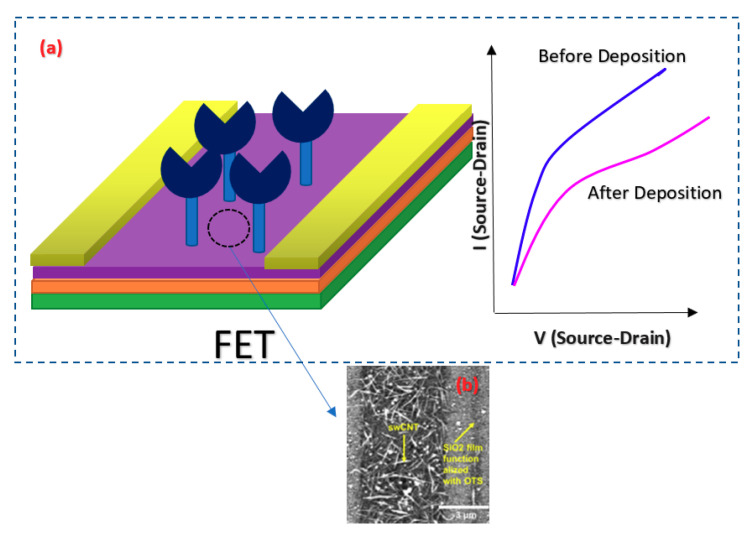
(**a**) Electrical identification of the viral particles using a tailor-made single-walled carbon nanotube (SWCNT)-FET immunosensor; (**b**) AFM image of the SWCNT channel region.

CNTs have been the focus of attention from researchers’ viewpoint due to their superior conductivity and exceptional configuration [132,133]. These hollow carbon-based arrangements might have one (SWCNT) or more walls (multi-walled CNT) consisting of hexagonal frames of carbon [134]. In other words, they are rolled graphene sheets that have been capped at both ends. Owing to their systematic and well-organized structure, they display high mechanical stamina, which makes them an ideal choice for biosensing applications [135,136]. In addition, CNTs deliver a broad surface area for immobilizing a great number of BREs [137]. Thus, they are being commonly employed in detecting virus-related biomarkers in biological samples. For instance, a FET-based assay was developed for recognizing tiny amounts of HCV employing SWCNT. PNA was used as the probe and detected 0.5 pM of the target particles [87]. In a different research work, a simple-to-use FET biosensor was projected for the quantification of aerosolized viruses (see Figure 22). The core strategy of this approach was the higher number of charge carriers and larger sizes of the antibody–antigen complexes compared to unbound antibodies or viruses. This fact led to the deposition of these big particles on a specific region of the substrate. In the case that this area overlaps with the SWCNT-modified channel of the transistor, a change will occur in the recorded current that signifies the existence of the virus [91].

#### 3.1.5. Transition Metal Dichalcogenides (TMDs)

The other category of surface functionalization materials is the TMDs [138]. They are single-layer semiconductors that are made up of two chalcogen atoms and a group IV, V, or VI transition metal component [139]. These single-layer hexagonal structures contain a direct bandgap that makes them suitable candidates for electronic applications [140]. One of the most popular two-dimensional TMDs—Molybdenum disulfide (MoS_2_)—has attracted attention in biomedical fields attributable to its superior electronic, chemical and mechanical characteristics [141]. This graphene-like arrangement has a width equal to a single cell, which results in its ultra-sensitive performance when used in biosensing platforms. They can be easily integrated into biosensors since they do not have any loose bond in their structure [142]. Today, they are broadly being employed especially in Chem/BioFETs [143]. For example, an accurate MoS_2_-FET genosensor was established for detecting Down syndrome. They modified MoS_2_ with gold nanoparticles in order to facilitate DNA immobilization. The device presented an ultra-sensitive performance by detecting down to 100 aM of the target DNA sequences [144]. Another easy-to-use and the precise system was designed using a MoS_2_-functionalized FET immunosensor for identifying Fibroblast growth factor 21 (FGF21)—a biomarker for fatty liver disease (NAFLD) detection. After introducing the sample containing the target analyte, and accordingly the production of Ab-Ag complexes, the detection occurred and a LoD of 10 fg·mL^−1^ was recorded. This device demonstrated a satisfactory selectivity even in complex serum samples [145]. A DNA biosensor based on a MoS_2_-based FET was introduced which employed phosphorodiamidate morpholino oligos (PMO)-DNA hybridization as their detection strategy. Negatively charged MoS_2_ was drop-casted on the positive-charge-baring sensing area and attached via electrostatic interactions. Next, the immobilization of PMOs took place, which enabled an ultra-sensitive detection. The low LoD (6 fM) of this device showed the outstanding capability of this structure in biosensing applications [146]. The use of TMDs in Chem/BioFETs is exemplified in these successful studies and many others [147,148,149,150], which confirms their high performance in detecting tiny biomolecules in complex human biofluids.

#### 3.1.6. Conducting Polymers (CPs)

CPs are one of the other appropriate choices for covering the surface of Chem/BioFETs since they are lightweight, cost-effective, highly scalable, easy-to-fabricate and have adjustable properties [151]. These functional materials have exceptional electrochemical characteristics, high electrical conductivity, mechanical stamina and thus are suitable for being used in biosensors as transducers [152]. Controlling their configuration and the use of dopants in the structure of CPs can further improve their performance by providing a large surface-to-volume ratio and boosted electrocatalytic behavior [153]. They can be utilized in the form of NWs or nanotubes on the sensing region of biosensors in order to enhance their sensitivity [154]. Modulating the CPs surface for optimized biomolecule linkages make their use in sensing platforms feasible [155]. For example, a very recent study reported the development of a conducting polymer nanotubes-functionalized FET aptasensor for dopamine (DA) recognition. Tailor-made aptamers were immobilized on the surface of carboxylated polypyrrole nanotubes (CPNTs), which resulted in the detection of as low as 100 pM of the DA. Controlling the diameter of CPNTs was an important feature, which the authors took into account while constructing this device. It was reported that the small structures demonstrated higher sensitivity and specificity in comparison to the wider ones since they delivered a large available area [156].

### 3.2. Different Types of Bio-Recognition Elements (BREs)

In order for Chem/BioFETs to have a precise and specific detection of antigen, antibody, nucleic acid, etc., their surface should be functionalized with tailored BREs. They operate based on the affinity between the antigen-antibody or DNA hybridization. Immobilizing specific BREs in their optimum arrangement enables the production of complementary complexes, which in turn change the conductance of the channel region. Generally, Chem/BioFETs can be categorized into three main groups of Immuno-, geno and apta-sensors. Antibodies against the viral/bacteria-related antigens, complementary DNA/RNA probes against the genomic material of the pathogen and tailor-made aptamers can be immobilized on the surface of modified FET devices in order to make them capable of capturing the desired target biomolecules.

#### 3.2.1. Antibody or Antigen

Antibody/antigen-immobilized FET-based biosensors -so-called immunosensors- are one of the most preferred systems for identifying either pathogen-specific antigens or produced antibodies in the host body in response to the pathogen attack. For example, the most recent research work for the identification of SARS-CoV-2 was carried out utilizing a FET-based biosensor decorated with ultra-selective antibodies to capture viral spike proteins. It could recognize as low as 100 fg/mL of the analyte in clinical transport medium [157]. A portable immunosensor was developed for sensing HIV-1. This single-molecule detection was carried out using an electrolyte-gated FET modified with antibodies against the capsid protein of HIV-1 p24 [105]. In another study, a GFET was designed using 1-pyrenebutanoic acid succinimidyl ester (PBASE) as the linker to immobilize specific antibodies for detecting the whole viruses. This strategy was successful enough to spot down to 47.8 aM of the target biomolecules [111]. The efficiency of these investigations and many others [89,91,92,93,103,106,107,108,110,115] acknowledge the capability of FET-based immunosensors in detecting infectious diseases.

#### 3.2.2. Nucleic Acid

Nucleic acid-based sensing systems or genosensors are the next widespread category of FET biosensors. The matching DNA or RNA of the virus, bacteria or any other pathogen is immobilized on the sensing area of the FET, and in the case that the target DNA/RNA is present in the sample, a signal is recorded as an indicator of the detection. For instance, a pioneering DNA biosensor was fabricated employing a SiNW-FET for HBV recognition. The probes were stabilized on the surface of (3-Aminopropyl) triethoxysilane (APTES)-functionalized nanowires. Due to the use of the rolling circle amplification strategy, a long strand of DNA was produced after introducing the targets. Therefore, an amplified electronic signal was recorded which enabled the detection of 1 fM of the target DNAs [90]. Another genosensing platform was proposed by Lee et al. They immobilized oligonucleotides on the surface of gold electrodes in order to detect the attachment of avian influenza virus sequences and achieved an LoD of 100 pM as seen in Table 1 [88]. In another effort, DNA was immobilized on CMOS-based ISFETs as BREs to detect *Plasmodium falciparum*. In this work, an adapted version of loop-mediated isothermal amplification (LAMP), called pH-LAMP, was employed, which enables ISFETs to detect pH variations during nucleic acid amplification. This CMOS-based device presented an outstanding performance, which shows its potential for being used as a PoC test of malaria [116].

#### 3.2.3. Aptamer

Aptamers are the other group of selective BREs, which have been used in several studies related to FET-biosensor. They are popular for ultra-sensitive sensing, particularly when they are incorporated with nanoparticles. As an example, an aptasensor was established for detecting HPV employing an rGO-FET. The pyrene molecules acted as linkers for the successful immobilization of RNA aptamers on the surface of the sensor. This real-time detection was able to sense down to 1.75 nM of HPV-16 E7 [109]. Another aptamer-functionalized Chem/BioFET was constructed to identify *Plasmodium falciparum* glutamate dehydrogenase in serum specimens. A specific aptamer was designed to capture the target pathogen precisely. An LoD of 48.6 pM was recorded that demonstrates this device holds the potential for detecting malaria even in asymptomatic patients [45].

#### 3.2.4. Other

Other types of BREs have been used for functionalizing Chem/BioFETs such as PNAs, sialoglycans, sialoglycopeptides, or even bacteriophages. For instance, an on-chip bacteria sensing system was reported, which used bacteriophages as bioreceptors for spotting a particular strain of *E. coli*. The potassium ions released from inside the bacterial cells due to phage infection was measured by a CMOS-based ISFET with polyvinyl chloride (PVC)-based potassium-sensitive membrane. This sensor could detect as low as 48.6 pM of the target biomolecules in below 30 min [22] (As seen in Table 1).

PNAs are the other novel type of BREs that have been used in a study for recognizing HCV. This SWNT-FET-based device was able to conduct a pM level detection [158]. Sialoglycans are also one of the desirable BREs that can be used in designing novel pathogen biosensors. They can mimic the natural host-cell surface and enhance the performance of the biosensor. For example, Ono and colleagues developed an influenza GFET-biosensing platform using sialic acid for coating the surface of the sensing region. Owing to the affinity of the virus’s HA molecules to this glycoprotein, the HA-sialic acid complexes are generated. Besides, neuraminidase (NA) interacts with these complexes, which result in the detection of the virus [113].

## 4. Readout Circuit and Systems

After preparation of the Chem/BioFET structure and modification and functionalization of the sensing surface according to the desired application, the parameters of Chem/BioFETs should be controlled and measured using electrical devices, which might be laboratory equipment like a potentiostat and a semiconductor parameter analyzer [87,89,91,94] or integrated circuits [20,22,23,88,116]. This section gives an overview of readout techniques. Herein, the main focus is on the CMOS-based integrated circuits, which can pave the way for the development of affordable and handheld PoC devices. If the Chem/BioFETs are adapted to CMOS technology, it would be possible to miniature them together with the circuits of the required signal amplifiers, filters, multiplexers and analog-to-digital converters (ADCs) on a single chip. CMOS technology offers the great advantages of small size, lightweight, low cost, fast response, high spatiotemporal resolution, low power consumption, good noise immunity and high level of integration.

Table 2 compares various readout circuits reported for Chem/BioFET sensors, especially CMOS-based ones. In 1999, Bausells et al. [159] focused on the fabrication of ISFETs in an unmodified CMOS process (standard CMOS process following the exact steps for MOSFET fabrication) and showed that it is possible to integrate sensing and electronic functions. They designed an integrated ISFET-amplifier circuit by employing standard cells from the CMOS process. Since many efforts have been made to fabricate ISFETs in CMOS technology, most of the circuits reported in Table 2 take advantage of ISFETs. Although many of them are used for pH measurement using ISFETs, they are potentially useful for reading the output parameters of Chem/BioFETs. Furthermore, as seen in both Table 1 and Table 2, ISFETs [20,22,23,88,116] are also useful for the detection of infectious agents. For instance, Rothberg et al. [20] presented a scalable CMOS-based ISFET sensor architecture for DNA sequencing of bacterial genomes including *V. fischeri*, *E. coli*, and *R. palustris* by measuring the pH variations during DNA sequencing. In other efforts, Malpartida-Cardenas et al. [116] and Lee et al. [88] reported CMOS-based ISFETs for the diagnosis of *P. falciparum* and H5N1 AIV through DNA sensing, respectively.

As seen in Table 2, Chem/BioFETs can be biased in different operation regions, which provide various types of readout circuits with different features. So, before introducing the readout circuits, it is required to take a look at the measurement principles in these operation regions.

As aforementioned, among various Chem/BioFETs, the equations of ISFETs have been theorized perfectly which were briefly reviewed in Section 2. The output current of ISFET in the linear region was expressed by Equation (1) where the threshold voltage follows Equation (5). However, Since the gate of ISFET is in contact with electrolyte with no metal in its vicinity, internal source and drain resistances (*R*_s_ and *R*_d_) are formed based on the actual geometry of the device in such a way that the actual drain-source voltage and the gate-source voltage of the ISFET are equal to *V*_DS,ISFET_ = *V*_DS_ − (*R*_s_ + *R*_d_)*I*_D_ and *V*_GS,ISFET_ = *V*_GS_ − *R*_s_*I*_D_ and, consequently, the sensitivity is declined [160]. A technological approach to solve this problem is shortening the length of the source and drain diffusions, which implies new technologies. However, using electronic readout circuits insensitive to series resistances is much easier. By biasing an ISFET at a constant *I*_D_ with a constant *V*_DS_, *V*_GS_ will adjust to compensate for a change in threshold voltage due to a pH variation [160].

**Table 2:** Different readout circuits for Chem/BioFET sensors.

CMOS Tech.	Array #	Diff.	Operational Region of FET	Configuration	Output Signal	Resolution	Sensitivity	IDR	Sensing Area (µm^2^)	Pixel/Active Area (µm^2^)	Total Area (mm^2^)	Power (mW)	Ref.
1.6 µm	1	No	Sat.	Wheatstone-bridge, variable *V*_Ref_	V	-	58 mV/pH	-	-	-	4 × 4	-	[161]
-	1	No	Tr.	CVCC	V	-	-	-	-	-	-	-	[160]
0.35 µm	1.5 M	-	-	CVCC	-	-	-	-	-	-	10.6 × 10.9	-	[20] *
	7.2 M										17.5 × 17.5		
	13 M										17.5 × 17.5		
1.0 µm	1	No	-	CVCC, Feedback	V	-	47 mV/pH	pH: 2.5 to 9.2	-	-	-	-	[159]
2.5 µm	-	No	Tr.	CVCC	V	-	58 mV/pH	pH: 3 to 11	-	-	<0.25	10	[162]
0.18 µm	6	No	Tr.	CVCC	V	-	-	-	-	-	1.5 × 0.6	-	[22] *
0.35 µm	64 × 64	No	-	CVCC, SPT	V	-	20 mV/pH	pH: 4 to 10	-	10.2 × 10.2	0.7158 × 0.7158	-	[163]
0.35 µm	64 × 64	No	Tr.	CVCC, APS	D	-	−9.23 mV/pH	-	-	96	0.56	-	[116] *
5 μm	10 × 10	No	-	CVCC, APS	V	-	−229 mV/pH	pH: 4 to 9.1	2000 × 2000 (total)	200 × 200	5.1 × 5.1	-	[164]
2 µm	1024 × 1024	No	-	CVCC, Charge transfer APS	V	-	29.8 mV/pH	pH: 2 to 10	-	23.55 × 23.55	14.8 × 14.8	-	[165]
0.35 µm	3 × 11	No	Sub.	CVCC, integrate-and-fire topology, AER	D	-	−7.73 dB/pH	pH: 1 to 14	57.5 × 57.5	80 × 100	-	(EP: 157 nW)	[166]
0.35 µm	8 × 4	No	-	CVCC, Feedback to the gate	V	60.3 mpH	42.1 mV/pH	pH: 1 to 14	-	60 × 70	2 × 2.5	SFE: 4.841 × 10^−4^	[167]
0.35 µm	1	No	Sat.	CVCC, Feedback, PG	V	-	200 mV/pH	-	30 × 100	-	0.6 × 0.5	-	[168]
0.18 µm	8 × 8	No	Sub.	CVCC, Current feedback	F	-	37 mV/pH	pH: 4 to 10	-	-	2.6	0.076	[169]
0.35 µm	1	No	-	CVCC, VCO	F	-	78 kHz/pH	pH: 0 to 7	-	-	0.045	0.12	[170]
0.35 µm	64 × 128	No	Tr.	CVCC, APS	D	-	−9.23 mV/pH	-	9.3 × 10.3	-	2×4	-	[171]
	64 × 200		VS	CM			−1.033 µA/pH		6.5 × 7.775				
	64 × 200		VS	CM, PG			−0.717 µA/pH		6.5 × 7.775				
-	1	No	Sub.	CM, Current feedback	V	-	−49.4 mV/pH	pH: 4 to 9	-	-	-	-	[172]
0.35 µm	3 × 3	No	Sub	CM, PG, RO	F	0.008 pH	6 to 8 kHz/pH	pH: 5 to 7	55 × 65	64 × 54	0.1089	6 × 10^−3^	[173]
0.35 µm	128 × 128	No	Tr.	CM, CC, Auto-zeroing, S/H	D	0.24 pH (@1000 fps) 0.45 pH (@3000 fps)	50 LSBs/pH	pH: 4 to 10	-	18 × 12.5	2.6 × 2.2	376	[174]
0.35 µm	8 × 8	No	Sub.	VM, PG, Optic., MM	D		57 mV/pH	pH: 4 to 10	-	-	-	-	[175]
0.18 µm	64 × 64	No	-	pH-TC, Optic.	D	-	−26.2 mV/pH (G = 1) −103.8 mV/pH (G = 4)	pH ~ 1 to 14	Chem.: 22.3 Opt.: 20.1	10 × 10	2.5 × 5	105.6	[176] *
65 nm	512 × 128	No	Sub.	pH-TVC	D	0.01 pH	123.8 mV/pH	pH: 2.5 to 11.5	15	4.4. × 4.4	512 × 128	PA: 80.6, AB: 108.4, DB: 6.5	[23] *
0.18 µm	8 × 8	No	Sub.	pH-TC, APS, PWM	T	33 mpH	6.1 μs/pH	-	-	16.5 × 16.25	6.7	8.3	[177]
0.18 µm	3 × 3	No	-	pH-TC	D	0.028 pH	27 ns/pH	-	10 × 10	-	0.036	0.23	[178]
0.35 µm	1	No	Sub.	ISFET logic	V	0.5 pH	3700 mV/pH	pH: 3.7 to 10.95	95 × 200	-	-	-	[179]
0.35 µm	8 × 8	No	Sat.	ISFET logic	V	0.5 pH	50 mV/pH	pH: 1 to 14	-	-	-	-	[180]
0.35 µm	4 pairs	Yes	Sat.	CVCC, PG, Feedback to the gate	D	-	100 mV/pH	pH: 5 to 9	30 × 100	120 × 120	0.65 × 0.5	-	[181]
0.35 µm	2 × 2	Yes	Tr.	ISFET/REFET diff., CVCC	D	-	40 mV/pH	-	11.6 × 11.6	-	1.4 × 2.6	15	[182]
0.35 µm	16 × 16	Yes	Tr.	ISFET/REFET diff., CVCC	D	-	46 mV/pH	pH: 3.28 to 7.22	11.6 × 11.6	12.8 × 12.8	-	60	[63]
0.6 µm	1	Yes	Sat.	ISFET/REFET diff., CVCC	V	-	400 mV/pH	6 pH	-	-	18.225	2.1	[183]
(CMOS)	1	Yes	-	ISFET/REFET diff.	V	0.01 pH	−40 to −43 mV/pH	pH: 4 to 9	-	1650 × 2600	4.9 × 3.9	-	[184]
2.5 µm	1	Yes.	Tr.	ISFET/REFET diff., CVCC	C	0.15 pH	−0.3875 µA/pH	pH: 3 to 11	-	-	-	-	[185]
0.35 µm	1	Yes	Tr.	ISFET/ISFET diff., CM	D	0.1 pH	800 mV/pH	pH: 5 to 9	-	-	-	-	[186]
0.35 µm	1	Yes	Sub.	Diff, Gilbert gain cell, CM, Translinear	C	-	45 mV/pH	pH: 5 to 9	34 × 100	-	2.5 × 2.81	1.65 × 10^−4^	[187]
0.35 µm	1	Yes	-	Diff, direct charge accumulation	D	36 µV	-	88.3 dB	71 × 71	1000 × 1640	4 × 5	-	[88] *

* The papers reported for the detection of infectious agents, VM: Voltage-mode, CM: Current-mode, VS: Velocity saturation, PG: Programmable gate, Tr.: Triode, Sat.: Saturation, PA: Pixel array, AB: Analog blocks, DB: Digital blocks, pH-TVC: pH-to-time-to-voltage converter, pH-TC: pH-to-time converter, Sub.: Subthreshold, IDR: Input dynamic range, F: Frequency, SFE: Single front end., CVCC: Constant-voltage constant-current, CC: Current conveyor, SPT: Standard pixel topology, APS: Active pixel sensor, AER: Address event representation, V: Voltage, D: Digital, C: Current, T: Time, EP: Each pixel, Opt.: Optical sensor, Chem.: Chemical, RO: Ring oscillator, S/H: Sample and hold, MM: Magnetic manipulation, G: Gain.

Linear operation mode is one of the operational regions that can be used in current-mode circuits. If the ISFET is biased in the triode region with a low *V*_DS_, the output current mentioned by Equation (1) can be approximated by [32]:

(9) $$I_{D}=\mu C_{\mathrm{ox}}\frac{W}{L}\left[\left(V_{\mathrm{GS}}-V_{t\left(\mathrm{ISFET}\right)}\right)V_{\mathrm{DS}}\right],$$

where *V*_t(ISFET)_ is dependent on pH based on Equation (5). So, *I*_D_ has linear sensitivity to pH [32]:

(10) $$\frac{dI_{D}}{dpH}=g_{m}\left(pH\right)=-2.3\alpha \mu C_{\mathrm{ox}}\frac{W}{L}V_{\mathrm{th}}V_{\mathrm{DS}},$$

The width of the channel (*W*) biased in the triode region is determined by the gain requirements but at the expense of the occupied area and array density. Moreover, to reduce the second-order sources of non-linearity (like mobility degradation) as well as short-channel effects, the length of the transistor (*L*) should be long enough.

The principle of measuring the threshold voltage of ISFET in the saturation region is similar to triode ISFET. Furthermore, some low-voltage low-power circuits are reported for the ISFETs operating in weak inversion, as a translinear element. Velocity saturation as a short-channel effect can also be used as a linear mode of operation for current-mode readout circuits. Considering *V*_t(ISFET)_ follows Equation (5), the sensitivity of *I*_D_ to pH is linear [32]:

(11) $$\frac{dI_{D}}{dpH}=g_{m}\left(pH\right)=-2.3\alpha V_{\mathrm{th}}v_{\mathrm{sat}}C_{\mathrm{ox}}W,$$

By comparing with Equation (10), it can be deduced that, in contrast to the triode region, the operation of the transistor in velocity saturation is insensitive to mobility (*µ*), *V*_DS_, and length (*L*) mismatches, especially when the minimum length is used. The transistor in velocity saturation naturally needs to be a short-channel device and small width is preferred to limit the biasing current [32].

The following subsections outline different circuits reported for measuring the outputs of Chem/BioFET.

### 4.1. Non-Differential Measurements

Many of the Chem/BioFET readout circuits involve non-differential measurement topology, which can have various configurations as described in the following subsections.

#### 4.1.1. Variable Voltage Reference in Feedback Mode

One technique is controlling the variations of *I*_D_ in a feedback loop and automatic adjustment of the potential of the RE, and thus of the liquid, with respect to the source and drain potentials. So, any change in *V*_t_ will be compensated by the variation of *V*_gs_ via the reference voltage of RE, *V*_Ref_.

**Figure 23:**
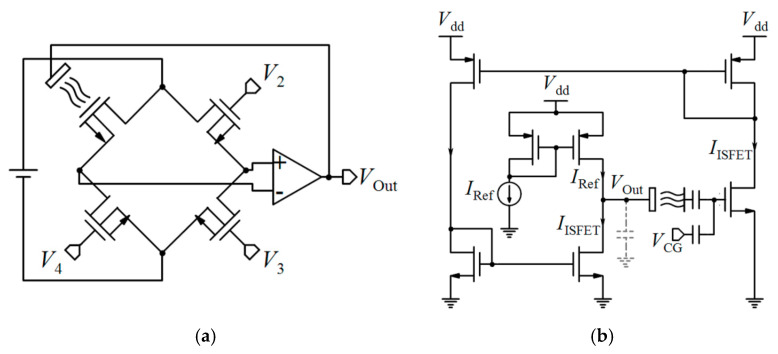
(**a**) Direct Wheatstone-bridge; (**b**) the circuit used for the characterization of ISFET drift in the feedback mode.

For instance, an automatically balanced bridge circuit [161] like Figure 23a can compensate a change in *V*_t_ by changing *V*_gs_, via the RE (see Table 2). However, this technique forces the liquid sample to a certain potential while the liquid may not be grounded. Moreover, the asymmetrical impedance of the source and drain leads the system sensitive to static electricity and electric fields [160]. Furthermore, this approach is not suitable for measuring multiple ISFETs with one RE [188].

Figure 23b shows a programmable gate (PG)-ISFET readout circuit proposed by Georgiou et al. [189] in which the current of ISFET is compared and set with a reference current, *I*_Ref_, in a feedback loop, and *V*_Out_ is adjusted by the effective gate-source voltage of the ISFET. In another effort, Jamasb et al. [190] reported another circuit in which the voltage reference is set in a feedback loop.

Since this biasing is not constant, an array structure with this configuration will require a distinct RE for each pixel leading to a complex setup. So, this technique is not a popular configuration [191].

#### 4.1.2. Constant-Voltage Constant-Current (CVCC)

CVCC configuration is the most popular one for the readout circuits of Chem/BioFET sensors in which Chem/BioFET current and drain voltage are constant and the source voltage varies with pH. This technique, which was proposed by Bergveld [160], is an automatic adjustment of the source and drain potentials in respect of a constant liquid potential which can be accidentally the ground potential in practice. So, the best way is to ground the liquid utilizing a grounded reference. Source/drain follower circuit can provide these conditions and is the most convenient approach to detect the variations of ISFET threshold voltage [160]. CVCC configuration is immune to capacitive scaling of the pH signal at the floating gate of the ISFET. Moreover, the arrays designed based on this technique are compact and only need a switch and an ISFET for each pixel [191].

**Figure 24:**
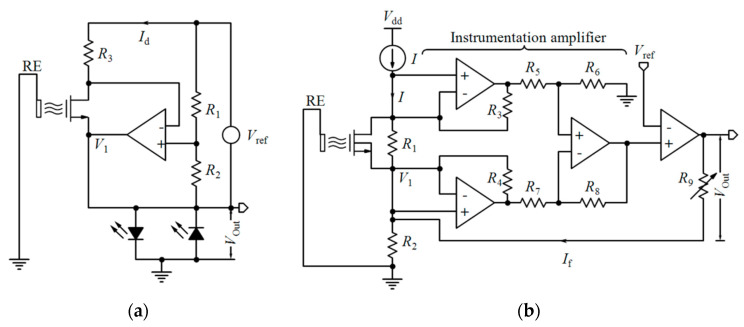
(**a**) A basic diagram of a source/drain follower readout circuit; (**b**) an ISFET amplifier proposed by Bergveld et al.

Figure 24a shows a simple version of a source/drain follower using unsaturated ISFET [188]. The operational amplifier provides equal input voltages and so *V*_R1_ = *V*_R3_ and *V*_R2_ = *V*_ds_, which are constant (*V*_R1_ and *V*_R2_ are parts of constant *V*_ref_). As a result, *I*_d_ = *V*_R3_/*R*_3_ is also constant. Thus, *V*_gs_ automatically adapt the source potential with respect to the ground to compensate the variation of ISFET threshold voltage, *V*_t_, and the output voltage accurately reproduces the changes in the oxide surface potential. The diodes at the output, which are practically light-emitting diodes (LEDs), provide a limitation of *V*_Out_, and thus the source voltage with respect to ground, and light up when the system is out of range.

Figure 24b shows another source/drain follower that uses the instrumentation amplifier concept and unsaturated ISFET [160]. The inputs of the instrumentation amplifier are connected to a fixed voltage, *IR*_1_ and its output voltage is inversely proportional to the channel resistance of the ISFET. An operational amplifier amplifies the difference between the output voltage of the instrumentation amplifier and *V*_ref_, which is an adjustable reference voltage. The output of this amplifier injects a feedback current, *I*_f_, into *R*_2_ and provides a constant *I*_d_ and a constant *V*_ds_ = *IR*_1_ by controlling the source voltage (*V*_R2_) and drain voltage (*V*_R1+R2_). *I*_f_ is measured via the adjustable resistor *R*_9_. The source and drain potential in respect of ground follow a change in the effective input voltage *V*_1_ whose amplified version is available across *R*_9_ and is equal to *V*_Out_ = (*R*_9_/*R*_2_)*V*_1_. The reference voltage, which should be independent of temperature, is adjusted in such a way that *I*_f_ = 0 when the ISFET is placed in a neutral buffer (pH = 7). A circuitry (not shown in Figure 24b) was also added for temperature measurement and compensation of temperature drift in *V*_gs_(*T*) for constant *I*_d_.

As reported by Hammond et al. [192], two source measure units (SMU1 and SMU2) on a parameter analyzer along with a feedback loop composed of a fixed resistor and two operational amplifiers can provide the bias condition of saturated ISFET and the RE can be biased at a suitable voltage by another SMU (SMU3). Nikkoo et al. [22] integrated a similar source/drain follower along with potassium-sensitive FET using CMOS technology for rapid detection of *E. coli* bacteria.

In another effort, Ravezzi et al. [162] used a structure employing cascode current mirrors with output impedance to provide the ISFET drain current with low dependence on its *V*_ds_. Three operational amplifiers were adapted to the structure, two of which biased the ISFET at the triode region and the last one operated in the follower configuration to de-couple the output terminal from the biasing circuit.

**Figure 25:**
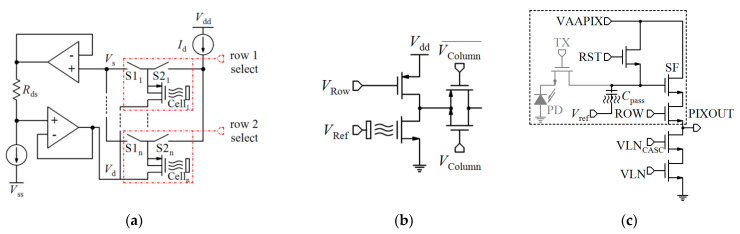
(**a**) Schematic of a column of 16 pixels in the *n* × *n*-array sensor chip; (**b**) a standard ISFET pixel readout; (**c**) a dual-pixel schematic including a CMOS image sensor and a source follower.

The infinite input impedance of a source/drain follower leads the circuit to not load the system during measurement. However, op-amps increase area occupation and power consumption. To decrease the power consumption and area occupation of array structures with a large number of pixels that employ op-amps, the readout circuit can be shared between pixels in the same row or column [63,182,183,193,194]. Figure 25a illustrates a single column of *n* half-cells in an *n* × *n* array. Milgrew et al. [63] used this architecture and reported a CMOS sensor chip including a 16 × 16 pixel array of ISFETs for direct extracellular imaging.

Rothberg et al. [194] improved FET pixel and array designs to achieve high accuracy and measurement sensitivity as well as small pixel sizes and dense arrays. Different pixel designs are proposed by this group based on both p-channel ISFETs and n-channel ISFETs. The same group [20] fabricated a chip with 1.5 M, 7.2 M and 13 M ISFETs (see Table 2) and used it for simultaneous detection of independent DNA sequencing of three bacterial genomes (*V. fischeri*, *E. coli*, and *R. palustris*).

There are also some other techniques that are based on CVCC method, but the output node of the circuit is connected to ISFET floating gate through a capacitor or switch. For instance, Hu et al. [167] designed a circuit using feedback and a low-leakage switching scheme and overcame the problems of transconductance reduction due to capacitive division and DC offset due to trapped charge. Chan et al. [169] also used an averaging array based on a global negative current feedback technique to regulate the undefined threshold voltages of ISFETs.

Some array structures [163,195] are designed based on a standard pixel readout like Figure 25b which is similar to CVCC and consists of an ISFET and two switches. In another effort, as mentioned in Table 2, Malpartida-Cardenas et al. [116] used a 64 × 64 array whose pixels were implemented as a source follower configuration and convert pH to voltage and employed this sensor for *plasmodium falciparum* malaria diagnosis and artemisinin-resistance detection.

Several papers integrated active pixel sensors (APS) using ISFETs by employing the ISFETs as the standard buffer [175,176] or the replacement of the photodiode by the ISFET [164,165] (by using a charge transfer technique). Huang et al. [176] proposed a dual-mode sensor by integrating a CMOS image sensor with the ISFET as the standard buffer to achieve accurate pH detection towards DNA sequencing. The dual-mode pixel is depicted in Figure 25c. The circuit shown in gray is added to an ISFET pixel to provide optical sensing along with pH measurement. The poly-gate of ISFET (SF) is connected to the top metal and Si_3_N_4_ passivation layer (as ion-sensitive membrane of ISFET). They also employed correlated double sampling to mitigate pixel-to-pixel non-uniformity like threshold voltage mismatch.

#### 4.1.3. Current-Mode Readout Circuits

Current-mode readout techniques [31,32,172,196,197] have also attracted the attention of researchers. In this approach, the output current of ISFET is measured, which can take the advantages of current-mode techniques and is also compatible with weak inversion operation, as well as linear and velocity saturation regions [191].

**Figure 26:**
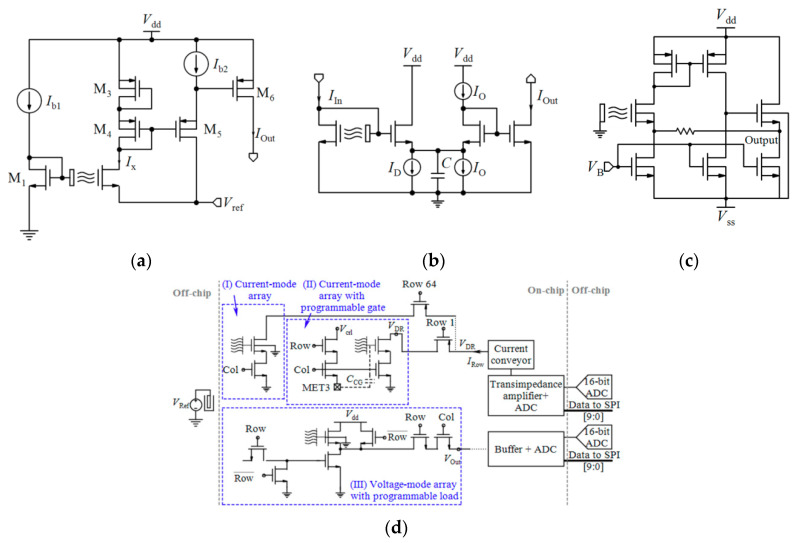
(**a**) HCell with the translinear operation; (**b**) a log-domain filter with an ISFET used at its input; (**c**) a current-mode readout with current feedback; (**d**) schematic of a system including three configurations: (I) Current-mode configuration in a 64 × 200 array, (II) current-mode PG pixel architecture in a 64 × 200 array, (III) voltage-mode pixel structure in a 64 × 128 array.

The weak inversion region provides drain currents in the order of nano-ampers and below that is suitable for designing low-power circuits. In addition, the region has the highest transconductance to current ratio and so the maximum intrinsic voltage gain [31]. Low *V*_ds_ required for saturation, minimum gate capacitance and low gate voltage in this region are the other advantages of weak inversion [65]. Moreover, the exponential relationship of *V*_gs_-*I*_d_ allows for using bipolar circuit techniques like log-domain filters and translinear circuits for ISFET applications leading to novel biochemical systems. For example, Figure 26a illustrates a hydrogen cell (Hcell) based on a translinear operation reported by Shepherd et al. [31,198] whose output current is directly proportional to hydrogen ion [H^+^] concentration. By setting *I*_b1_ = *I*_b2_ and considering *K*_chem_ = *exp*(−*γ*/*nV*_th_) and Equation (7), it can be proved that [31]:

(12) $$I_{\mathrm{Out}}=I_{b1}exp\left(\frac{-2V_{\mathrm{ref}}}{nV_{\mathrm{th}}}\right)K_{\mathrm{chem}}^{2}\left[H^{+}\right],$$

As another technique, Georgiou et al. [197] presented a log domain chemical filter utilizing an ISFET biased in weak inversion. Figure 26b shows a log-domain filter with an ISFET used at its input. The filter parameters like gain and cut-off frequency can be dependent on the ion concentration of the analyte.

An array configuration was used by Miscourides et al. [32] in which direct controlling of the *V*_ds_ of each device gets access to an individual pixel. Each drain row is connected to a generic current conveyor (CC), which leads the voltage of that row to be constant at *V*_DR_ and guarantees the generation of an output current depending only on *V*_gs_ during readout of each pixel. A 128 × 128 current-mode ISFET array with in-pixel calibration is proposed by Zeng et al. [174], which takes advantage of a CC, auto zeroing technique and sample and hold (see Table 2). In another effort, Pookaiyaudom et al. [199] embedded a CC type 2 (CCII^+^) with two ISFETs as its input transistors.

A configuration was used by Miscourides et al. [32] in which the ISFETs were biased in the velocity saturation region and includes a pixel switch to turn on only one pixel at any time. The higher drain current in the velocity saturation region in comparison to the triode region would make it unsustainable to simultaneously output the currents of all column pixels (see Equations (10) and (11)). To compensate the offset, the same group [200] extended their current-mode array to include a PG inside each pixel. As a continuation of these works, this group compares these two configurations with a voltage-mode source-follower configuration. Figure 26d shows the schematic of a system including a current-mode configuration in a 64 × 200 array, current-mode PG pixel architecture in a 64 × 200 array, voltage-mode pixel structure in a 64 × 128 array, current conveyor, transimpedance amplifier, buffer, two on-chip and two off-chip ADC. The two current-mode configurations showed better linearity and smaller size than the voltage-mode circuit and they concluded that the current-mode approach provides the best overall performance [171].

An integrate-and-fire topology with address event representation (AER) was used by Georgiou et al. [166] to encode the pH-sensitive current in frequency. Despite its low power consumption (157 nW at pH = 7), the signal processing using specific ADCs in the spike domain makes this technique complex. The same group [173] used a similar current-mode pixel architecture along with an averaging mirror for robust measurement and a ring oscillator for converting the current to frequency.

In some current-mode circuits [172,201,202], a current feedback is used for stable DC operation. For example, Figure 26c shows a current-mode readout with global feedback proposed by Premanode et al. [172].

**Figure 27:**
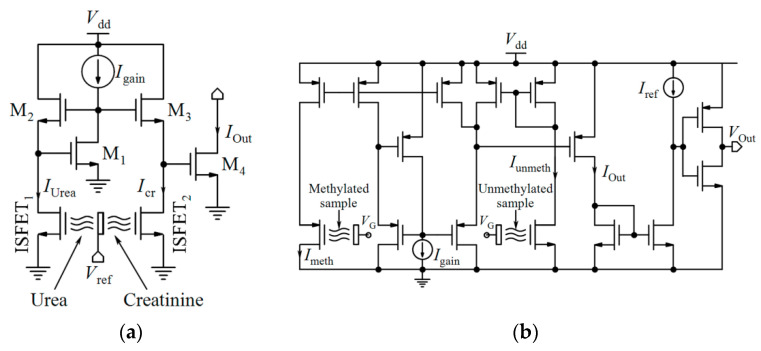
Ratiometric measurement: (**a**) ISFET based translinear divider; (**b**) a translinear circuit proposed by Kalofonou et al.

Juffali et al. [203] proposed another circuit based on translinear operation employing a division circuit and two ISFETs that can directly measure the ratio of urea to creatinine (an important marker for detection of pre-renal failure). Figure 27a illustrates the ISFET based translinear divider. In another effort, Kalofonou et al. [204] proposed a ratiometric approach of DNA methylation using ISFET in a translinear circuit (as depicted in Figure 27b). This sensor can determine the level of aberrancy of methylation between unmethylated and highly methylated genes and identify early signs of human neoplasias through blood circulation.

#### 4.1.4. ISFET Logic

In 2009, Wong et al. [205] proposed discrete ISFET logic for the detection of chemical reaction threshold which is useful for applications like detecting a specific gene in a DNA sequence where there is no need to measure the exact value of pH. For example, the switching threshold of an inverter can give a yes/no answer based on the pH variations. Other components like a switch [179,206], Schmitt trigger [207] or a NAND gate [40] can also be used as comparators.

**Figure 28:**
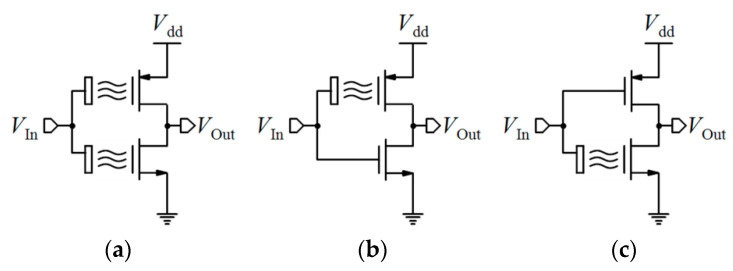
Three configurations of ISFET inverters: (**a**) p-type and n-type ISFET sharing the same membrane; (**b**) p-type ISFET with membrane and n-type MOSFET; (**c**) n-type ISFET with membrane and p-type MOSFET.

Figure 28a–c illustrates three configurations of an ISFET inverter with only one n-type or p-type ISFET or a pair of both. For example, Al-Ahdal et al. [179,206] used a complimentary pair of p-type and n-type ISFETs sharing the same ion-sensitive membrane as shown in Figure 28a and formed an ISFET-based chemical switch. Nabovati et al. [180] these switches in each pixel of an 8 × 8 array and achieve a wide pH range from 1 to 14 and the sensitivity of 50 mV/pH (see Table 2).

#### 4.1.5. pH-to-Time Conversion

**Figure 29:**
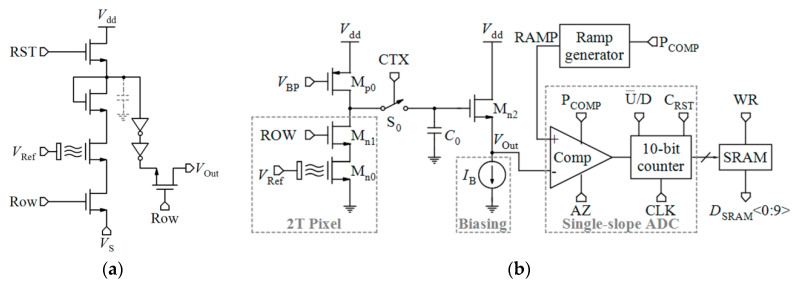
(**a**) An active pixel sensors (APS) based pH-to-time converter; (**b**) a readout circuit comprising a pH-to-time-to-voltage conversion (pH-TVC) amplification circuit and 10-bit voltage digital converter.

An mentioned above, the inverter with complementary ISFETs [208,209] can be the core of a compact pH-to-time converter capacitively coupled to a triangular wave whose switching point and, consequently, its output pulse width is modulated with pH. Pulse-width modulation (PWM)-based topologies is one of the suitable structures that allow in-pixel quantization and thus improve the SNR [191]. Wang et al. [178] proposed an ISFET pH-to-time sensor integrated with a fine on-chip time-to-digital converter (TDC) as the ADC with a thermometer-to-binary coder and a delay line. In another effort, Moser et al. [177,210] reported a pH-to-time converter based on APS as depicted in Figure 29a. This circuit modulates pH to pulse-width by using a periodic reset scheme and discharging a capacitor through the ISFET.

Jiang et al. [23,211] proposed a subthreshold pH-to-time-to-voltage conversion (pH-TVC) readout structure by employing pixel-level calibration and digital double sampling. As a result, the sensor can mitigate non-ideal effects, like passivation attenuation, parasitic capacitance and trapped charge. As demonstrated in Figure 29b, M_n0_ and M_n1_ are an ISFET and a row-selected transistor, and the other parts are shared by pixels in each column. M_p0_ and S_0_ (including complementary NMOS and PMOS transistor pair) are a pre-charge switch and a transfer switch, respectively. *C*_0_, the source follower M_n2_ and current source *I*_0_ are, in turn, used as integrator, buffer and the biasing circuit. A ramp generator, a single-slope ADC and an SRAM group are also utilized along with the pH-TVC readout circuit. Any change in pH results in a shift in the *V*_Out_. It can be proved that Δ*V*_Out_ is obtained by:

(13) $$\Delta V_{\mathrm{Out}}=\left(\frac{\Delta t}{C_{0}}\right)\cdot G_{m}\cdot A\cdot \Delta V_{\mathrm{chem}},$$

where *G*_m_, Δ*t*, Δ*V*_chem_, and *A* stand for the ISFET transistor transconductance, the charge integration time, pH-related potential change, and the attenuation factor, respectively. The amplification factor, (Δ*t*/*C*_0_)*G*_m_, is used to compensate the passivation sensitivity attenuation effect. This group [23] used this sensor for *E. coli* detection and achieved the sensitivity and resolution of 118.7 mV/pH and 0.01 pH, respectively (see Table 2).

### 4.2. Differential Measurements

Several differential architectures have been developed that measure the difference of the output signals of two ISFETs with the same REs. Differential techniques offer the advantages of temperature and long-term drift compensation as well as the elimination of common-mode noise. Moreover, these techniques can solve the problems of the unstable electrolyte-RE potential and the instability caused by the membrane layers of the ISFETs.

**Figure 30:**
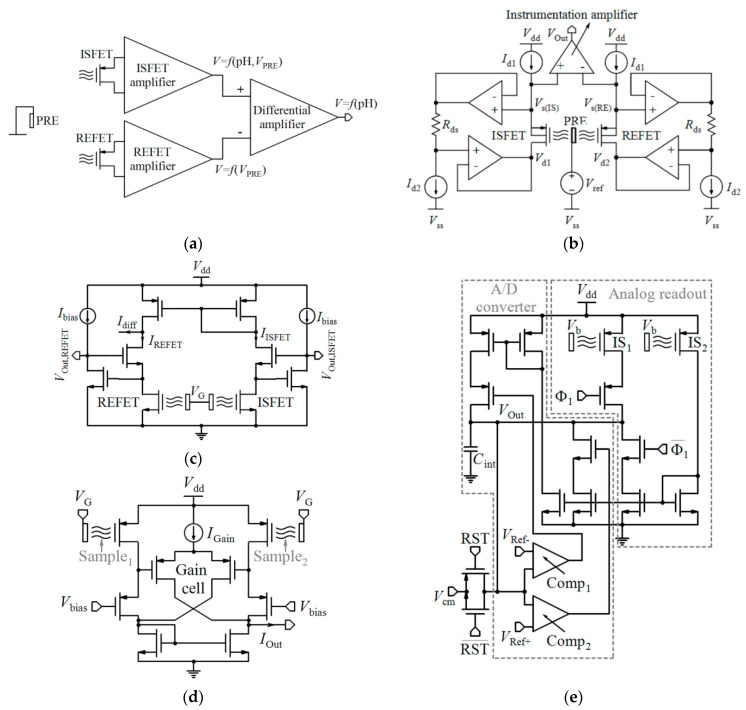
(**a**) A differential ISFET/reference FET (REFET) measuring system; (**b**) a differential pH-measurement pixel with ISFET and REFET for array structure; (**c**) ISFET/REFET voltage-clamped configuration; (**d**) a chemical Gilbert cell; (**e**) a ΣΔ ISFET readout circuit using differential measurement.

Lack of a solid-state RE is one of the issues in the field of solid-state chemical potentiometric sensors because they need to be combined with a liquid-filled RE whose stable lifetime is limited. Thus, in practice, a small sensor probe and a rather large RE are used together restricting the possibility of measuring in small sample volumes. For this reason, many research efforts have been dedicated to a differential measurement between an ISFET and an identical reference FET (REFET), which has a very low (ideally zero) sensitivity to the ion concentration under measurement. In this case, a metal wire can be used for grounding the sample solution [188]. So, a complete integrated ISFET-based sensor can be implemented by a differential structure composed of an ISFET and a REFET biased by a common counter-electrode as a pseudo-reference electrode (PRE) (Figure 30a) [188]. In this arrangement, the conventional RE is replaced by a PRE made of a noble metal, such as gold and platinum, to define the potential of the electrolyte without the need for any external RE. Covering the gate oxide of an ISFET within an additional ion insensitive membrane like a polymer [212,213] or chemical modification of the original gate oxide surface sites [214] are the most popular techniques to create a low-sensitive REFET. For practical reasons, bulk and source connections are usually short-circuited [213]. However, Bergveld et al. [46], in their seminal works, used bulk feedback to control the depletion charge in the bulk. Moreover, drift and different electrical properties of REFET and ISFET are some of the issues of this technique [193,215,216]. The DC bias of REFET and ISFET should be the same in such a way that they behave electrically identical. Chudy et al. [217] proposed to use a modified ISFET with additional membranes (CHEMFET) of which the outer membrane comprising a buffer component for the ion measurement. In a differential CHEMFET/REFET system, the electrically identical REFET with the same membrane does not contain such a buffer.

In 1989, Wong and White [184] proposed a CMOS-integrated op-amp for pH measurement which had an ISFET input and a feedback loop to a MOSFET. Then, they differentially amplified the outputs of two ISFET operational amplifiers with a PRE. In this primary implementation, a special coating on the gate of ISFETs was used to achieve different sensitivities and an ISFET submerged in a buffer solution of known pH played the role of a reference device. One of these ISFET-op-amps was composed of an ISFET with a Ta_2_O_5_/SiO_2_ gate and the other one included a SiO_x_N_y_/Si_3_N_4_/SiO_2_-gate ISFET with the sensitivity of 58–59 mV/pH and 18–20 mV/pH, respectively. The whole device could provide 40–43 mV/pH sensitivity and cancel common temperature effects as well as drift in both ISFETs (see Table 2).

Chodavarapu et al. [218] proposed another differential architecture for pH measurement including a gold PRE and a pair of ISFET operational transconductance amplifiers (IOTAs), each composed of an ISFET as its input. The ISFETs used in both IOTAs are identical, but different sizes of the p-MOSFET loads used in IOTAs cause variations in the sensitivity of these two IOTAs (50 mV/pH and 30 mV/pH). They could achieve a sensitivity of 40–45 mV/pH.

Hammond et al. [193] reported a system-on-chip (SOC) digital pH meter containing on-chip programmable voltage references and multiplexers controlled by a specially designed 8-bit microcontroller for use in a wireless diagnostic capsule and achieved a sensitivity of 37 bits/pH within an operating range of 7 pH units. Figure 30b shows the analog part of this differential ISFET/REFET pH-measurement circuit with a common PRE that includes two source-drain-follower configurations for the ISFET and REFET whose outputs are given to an instrumentation amplifier. In another effort, a similar differential structure was presented by Palan et al. [185] comprising two sources/drain follower circuits and a voltage-to-current converter (V/I). ISFET_1_ and ISFET_2_ were composed of Si_3_N_4_ and SiO_2_ ion-sensitive layers with the sensitivity of 52–58 mV/pH and 24–36 mV/pH, respectively, and implemented in a 2.5 µm CMOS technology. The simulation results showed that this interface circuit could provide a precision of ±0.15 pH (see Table 2).

An example of a pseudo-differential ISFET-REFET topology is the voltage-clamped circuit showed in Figure 30c [219], which can provide a fixed electrical bias and can operate in either weak or strong inversion. In another effort, Sohbati et al. [220] implemented an ISFET/REFET sensing front-end as part of an input differential pair. The same group [221] also proposed a pH-to-digital converter using two current comparator-based circuits including ISFET and REFET in each of which the output node is connected to the floating gate of FET. In another work, Liu et al. [181] proposed a differential topology based on the CVCC principle controlled with feedback to the floating gate of ISFET.

A ΣΔ ISFET readout circuit using differential current-mode measurement is also reported by Nabovati et al. [186], which is depicted in Figure 30e. The ISFETs depicted in Figure 30e operate in the linear region and the circuit works in three phases: (1) Reset, (2) evaluation, and (3) sampling. During the reset phase activated by RST and $\bar{\mathrm{RST}}$, the value of *C*_int_ capacitor is set to the common-mode voltage, *V*_cm_. During the evaluation phase, the clock Φ_1_ is activated and the ISFET current charges the *C*_int_. Combining Equations (1) and (5), we can deduce that the ISFET current in this region is proportional to the pH value. The current differential scheme increases sensitivity. The second ISFET (IS_2_) is a reference whose pH value is held at 7. In the sampling phase, the circuit is connected to the output for sampling. A charge to digital converter converts the output voltage to digital.

As another technique, a chemical Gilbert cell is demonstrated in Figure 30d. The differential pair includes two ISFETs that can be considered as a chemical translinear circuit [187]. This circuit is appropriate for applications that require differential monitoring in small volumes and several chambers, like studying more than one gene in DNA detection.

Lee et al. [88] proposed a CMOS device that directly measures the electrostatic induction of molecular charges in the working electrode with improved SNR and without any conversion loss. As seen in Figure 14, both working electrodes are connected to a voltage buffer circuit with high input impedance (due to employing a transistor with an isolated gate) and their voltages are within the operating range of the buffer circuit. The buffer transfers the induced charges in working electrodes to a fully differential switched-capacitor integrator followed by an ADC for the accumulation and digitization of the signal.

### 4.3. Amelioration of Some Non-Idealities

Trapped charges, noise and temperature sensitivity are some of the non-idealities that exist in ISFET measurements. This sub-section reviews some solutions to deal with them.

#### 4.3.1. Trapped Charges

The accumulation of trapped charge on the gate and passivation layer is one of the non-idealities that occurs during the fabrication of floating gate ISFETs in the standard CMOS process and causes a large unpredictable change in the threshold voltage of ISFET. UV radiation and bulk substrate biasing are the most convenient methods for the removal of the excess charge. The UV radiation can reach the inner polysilicon through a small aperture formed on top of the ISFET and excites the trapped charge that charges into the bulk region. This technique does not need any additional circuitry and can be employed for matching the electrical characteristics of ISFET pixels in an array structure. By the elimination of the mismatch between pixels threshold voltage, a single RE can be utilized for biasing the entire array [222].

Another technique is to use hot-electron injection, which can be adapted for threshold voltage programming. The operating point of ISFET can be calibrated by adding the electron to the floating gate and raising the threshold voltage [223]. However, this technique cannot decline the threshold voltage. Thus, electron tunneling is proposed by Al-Ahdal et al. [224] to eliminate electrons from the floating gate of the ISFET.

Georgiou et al. [225] reported another approach to program the floating gate of the ISFET by using a capacitively coupled floating gate, which allows the tenability of the operating point and the operation of the device within a tolerable gate voltage range by tuning the operating point. Yan et al. [168] also used two extra programmable nodes driven by a feedback configuration. Another technique is using a switch for extra connections to the floating gate of the ISFET in a feedback loop [167].

#### 4.3.2. Noise

The noise performance of the ISFETs is another non-ideality that can directly affect the sensor accuracy and resolution. The dominant low-frequency noise is 1/f noise. The measured noise characteristics in [226] showed that the Si/SiO_2_ interface dominates the noise behavior and the interface between the gate insulator and the solution does not contribute to measurable noise. To reduce the noise, the ISFET’s based on p-MOSFET can be used rather than n-MOSFET, because it is well established that the noise of p-MOSFET is lower by nearly two orders of magnitude [227]. However, n-type ISFETs have higher mobility and better drift performance.

#### 4.3.3. Temperature Sensitivity

Another factor affecting the performance of the ISFET is temperature sensitivity. Circuit compensation techniques can be used for the alleviation of temperature variations.

Wong et al. [184] proposed to use a differential configuration of an operational amplifier to match the ISFET sensor with a MOSFET and eliminate the temperature sensitivity. Equal electrical biases of the two devices are required to both devices have similar temperature characteristics, which might be difficult to be acquired by a differential pair comprising an ISFET and a MOSFET on the same chip [160] because of the differences in *V*_t_ leading to different bias conditions.

In another technique [228], the ISFET temperature coefficient, which is dependent on the operating point, is cancelled out by choosing a proper bias condition and empirical determination of a thermal operating point. Simultaneous detection and use of the parameters of the measuring device that cause the temperature drift is an unusual approach in conventional electronics. Bergveld et al. [160] used the feedback system of Figure 24b and also inject a sinusoidal signal by employing a transformer connected between the bulk and the source of the ISFET to adjust a certain bias condition and produce sensitivity independent of *β* = *µ*C_ox_ (*W*/*L*) and internal source and drain resistances. Sohbati et al. [221] used a periodic gate reset to accurately set the DC operating point of the ISFET.

Chin et al. [229] utilized a p-n diode as a temperature sensor whose negative temperature coefficient can cancel out the positive temperature coefficient of the ISFET by employing a summing circuit. Chung et al. [230] proposed another technique by using a threshold voltage extractor circuit to compensate for the ISFET thermal characteristic.

## 5. Conclusions

People’s health has been always endangered by infectious diseases such as universal viral outbreaks. Considering their significant impact on the fabric of society and the economy in general, the need for an expedited diagnosis for infectious agents (like viruses, bacteria, etc.) to control and limit its further spread into the population is inevitable in every country. We have learned expensive lessons from SARS-CoV-2 pandemic during which there was always a lack of systematic population testing methods by which, on one hand, the human interactions are minimized (a factor that extremely helps the government to curb the spread of disease very fast) and on the other hand, the infected people are treated at early stages at home and not when the situation is out of control and needs hospitalization. Therefore, surging toward the development of the extremely fast, reliable, portable, accurate and economical testing methods for fast detection of infectious agents’ fingerprint in the human body is of imperative importance. In this review, however, a detailed demonstration of different FET sensors dedicated to biosensing by precise looking at the pros and cons of every single design has been provided. Furthermore, bio-recognition elements at the surface of these sensors (see part 3, Table 1) have been also reviewed and the corresponding structural and bioreceptor functionalization of them are discussed. We have tried to bring all elements of FET-based biosensors dedicated to specific bio-species detection, which enables one to assess the physical structure, bio-recognition surface and circuit design of new concepts.

The study on the structural development of FET-based biosensors reveals that relying on the CMOS-based FET sensors enable the further standardization with well matured CMOS circuitry and mass production when scalability of the design is a concern. It has been discussed that integration of Chem/BioFETs with microfluidic chips is a stumbling block in front of successful integration of device with extremely small CMOS-based sensors. However, to address this inherent issue, there has been a tendency to develop extended gate structures for this purpose. Extended gate allows to have more sensing area, which can be integrated with microfluidic chip off the sensing area. Additionally, extended gate FETs would enable us to coat the sensor with novel nanomaterials such as graphene and carbon nanotubes to increase sensitivity and LoD with significantly improved response time. Another arrangement to take advantage of bigger sensing area and customized ion sensitive membrane is using floating membrane structure, which has the privilege of being based on the standard CMOS technology for making the gate off the oxide layer on the conductive channel. To have a better control over the sensitivity of the device, incorporation of double-gate and floating control-gate structures are recommended by researchers.

Another spotted avenue for detecting infectious agents using FET structures was using novel nanomaterials like carbon-based and other 2D nanomaterials. Granting that moving toward CMOS technology encompasses various merits that have been discussed above, however, in terms of sensitivity, it appears that nanomaterials like graphene, MoS_2_, SiNW, and CNTs would contribute much more. For instance, recently for COVID-19, a detection graphene-based single-layer FET has been proven to reach fM detection of the virus antibody. The reason seems to not be laid under the structure of the device, yet the inherent conductive channel electron transporting characteristics take the responsibility that arise from the superior surface chemistry of novel nanomaterials when they are in interaction with solution ions and biomolecules. Even though these nanomaterials provide a much faster response time and an extraordinary lower LoD compared to SiO_2_/Si_3_N_4_-on-silicon (which is being used in CMOS-based ISFETs fabrication), there are many scaling issues in the fabrication and integration of these sensors that need much more research and future studies.

The infection agent detection can be accomplished by label-free electrical detection of the agent itself, its specific antigens, nucleic acids fingerprint and also antibodies associated with that on FET-based biosensors. Using the multiplexed structure of sensors integrated with microfluidic channels could realize a combination of the detection scenarios mentioned above, which could offer a more robust and reliable design for detection of the targeted infectious agent. While there have been many success stories in published papers, the real-world realization of FET biosensors has a long way to be reliable. For instance, in human samples, there is a myriad of various proteins that in a non-specific attachment on the surface of FET sensor would create a false-positive response or true negatives that hamper the successful translational application of these sensors. Many efforts have been made to overcome the noises associated with unwanted attachment of bio-elements however they are done in very ideal laboratory conditions and very difficult to be used for real human samples. There are numerous hurdles in front of this technology if we want to replace the current gold standard like PCR with them for the detection of infectious agents. Many more studies are needed on the structural improvements, surface modification addressing the surface chemistry challenges and novel circuit designs that can reduce the noises associated with non-specific attachments and device inherent noises.

## Figures and Tables

**Table 1:** Recently proposed FET biosensors for detecting various infectious agents.

Application	Target	BRE	Linker	Surface	Detection Range	LoD	Sample	Ref.
IV diagnosis	GST-tagged-HA	CMP-NANA	APTES GA	SiNW	-	1 fM	Buffer	[102]
AIV diagnosis	Whole virus	Ab	MPTMS DTT biotin-HPDP	SiNW	-	10^−17^ M	Buffer	[103]
HBV diagnosis	HBV	ssDNA	Au	ITONWs	1 fM–10 μM	1 fM	Buffer	[94]
HBV diagnosis	DNA	DNA	APTES EDC/NHS	SiNW		1 fM	Buffer	[90]
AIV diagnosis	oligonucleotide	ssDNA	Thiol chain mercaptohexanol	Al/Au	0.1–100 nM	100 pM	Buffer	[88]
AIV diagnosis	HA protein	DNA Aptamer	-	Au microelectrode	10 pM–10 nM	5.9 pM	Chicken serum	[104]
HIV-1 diagnosis	capsid protein	Ab	EDC/NHS	Au	-	30 × 10^−^^21^ M	Serum	[105]
HBV diagnosis	Whole virus	Ab	GA	GNR	0.05–0.055 fM	0.05 fM	Buffer	[106]
Rotavirus diagnosis	Whole virus	Ab	PSE	MrGO	10–10^5^ pfu/mL	102 pfu	Buffer	[107]
Rotavirus diagnosis	Whole virus	Ab	pyrene-NHS	rGO	101–106 particle/mL	1 nm	Buffer	[108]
HPV diagnosis	E7 protein	RNA aptamer	EDC/NHS pyrene	rGO	30–1000 nM	1.75 nM	saliva	[109]
EVD diagnosis	glycoprotein	Ab	GA	rGO	-	1 ng·mL^−1^	Buffer, human serum, and plasma	[110]
EVD diagnosis	glycoprotein	Ab	PASE	rGO	2.4 × 10^−^^12^–1.2 × 10^−^^7^ g·mL^−1^	2.4 pg·mL^−1^	Spiked serum	[93]
VSV, MLV, HIV diagnosis	Whole virus	Ab	PASE	PET/PS/Graphene	47.8 aM–10.55 nM	47.8 aM	Buffer	[111]
Detection of HIV-1 viremia	RNA	?	-	SiO_2_/Si_3_N_4_	>1000 copies·mL^−1^	10 copies per reaction	Plasma samples	[21]
IV diagnosis	Whole virus	Sialoglycan	-	Graphene	-	2.56 HAU	Saliva	[112]
IV diagnosis	HA	SGP	PBASE	Graphene	-	200 nM	Buffer	[113]
COVID-19 diagnosis	S protein and whole virus	Ab	PBASE	Si/SiO_2_/Graphene	1.6 × 10^1^–1.6 × 10^4^ pfu/mL	2.42 × 10^2^ copies·mL^−1^	Clinical samples	[18]
HCV diagnosis	RNA	PNA	-	SWCNT	-	0.5 pM	buffer	[87]
virus detection	aerosolized bacteriophage MS2 and IV (H1N1)	Ab	-	OTS SAM SWCNT	-		Buffer	[91]
AIV diagnosis	Whole virus	sialic-acid-containing glycans	APTES LCEE	sialyllactose	10^0.5^–10^8.5^ TCID50/mL	10^0.5^ TCID50.mL^−1^	nasal mucus	[114]
AIV diagnosis	nucleoprotein	Ab	APTES EDC/NHS	SnO_2_	10^2^–10^5^ EID50/mL	10^3^ EID_50_ mL^−1^	cloacal swab	[92]
Zika virus diagnosis	Whole virus	Ab	-	AlGaN/GaN/disposable cover glass	0.1–100 ng·mL^−1^	0.1 ng·mL^−1^	Buffer	[115]
AIV diagnosis	AIa	Ab	SBP	CYTOPTM and Si_3_N_4_	10 fg·mL^−1^–100 pg·mL^−1^	1.9 fM 0.19 pM	Buffer	[89]
*Plasmodium falciparum* diagnosis	Nucleotide	DNA	-	SiO_2_/Si_3_N_4_	-	1 copy per reaction	Buffer	[116]
*Plasmodium falciparum* diagnosis	*Pf*GDH	Aptamer	MCH	IDμE	100 fM–10 nM		Serum	[45]
*E. coli* diagnosis	K^+^	Bacteriophage	-	PVC-based potassium-sensitive membrane	-	48.6 pM	Buffer	[22]

Ab: Antibody, GST: glutathione S-transferase, HA: Hemagglutinin, CMP-NANA: Cytidine-50-monophospho-*N*-acetylneuraminic acid, APTES: (3-Aminopropyl) triethoxysilane, GA: Glutaraldehyde, STV: streptavidin, AIV: Avian influenza virus, AIa: Avian influenza antigen, SBP: Silica binding protein, ssDNA: Single-stranded DNA, HBV: Hepatitis B virus, ITONWs: Indium tin oxide nanowire, EDC: *N*-ethyl-*N*′-dimethylaminopropyl carbodiimide, NHS: N-hydroxysuccinimide, MPTMS: 3-mercaptopropyltrimethoxysilane, Biotin-HPDP: N-(6-(biotinamido)hexyl)-3′-(2′-pyridyldithio)-propionamide, DTT: Dithiothreitol, HPV: Human papillomavirus, GNR: Graphene nanogrid, EVD: Ebola virus disease, HCV: Hepatitis C Virus, PNA: Peptide Nucleic Acid, LCEE: L-cysteine ethyl ester, PASE: 1-Pyrenebutanoic acid succinimidyl ester, PS: Polystyrene, VSV: Vesicular stomatitis Indiana, MLV: Murine leukemia virus, HIV: Human immunodeficiency virus, MrGO: Micropatterned reduced graphene oxide, PSE: 1-pyrenebutyric acid N-hydroxysuccinimide ester, SGP: Sialoglycopeptide, OTS SAM: Octadecyltrichlorosilane self-assembled monolayer, *Pf*GDH: *Plasmodium falciparum* glutamate dehydrogenase, MCH: Mercapto 1 hexanol, IDμE: Inter-digitated gold microelectrodes, PVC: Polyvinyl chloride.
